# Effectiveness of Interventions on Work Outcomes After Road Traffic Crash-Related Musculoskeletal Injuries: A Systematic Review and Meta-analysis

**DOI:** 10.1007/s10926-024-10185-z

**Published:** 2024-04-05

**Authors:** Charlotte L. Brakenridge, Esther J. Smits, Elise M. Gane, Nicole E. Andrews, Gina Williams, Venerina Johnston

**Affiliations:** 1 RECOVER Injury Research Centre, The University of Queensland, Brisbane, QLD Australia; 2 School of Human Movements and Nutrition Sciences, The University of Queensland, Brisbane, QLD Australia; 3 School of Health and Rehabilitation Sciences, The University of Queensland, Brisbane, QLD Australia; 4 Physiotherapy Department, Princess Alexandra Hospital, Brisbane, QLD Australia; 5 Centre for Functioning and Health Research, Metro South Health, Brisbane, QLD Australia; 6 Occupational Therapy Department, Metro North Hospital and Health Service, The Royal Brisbane and Women’s Hospital, Herston, QLD Australia; 7 Tess Cramond Pain and Research Centre, Metro North Hospital and Health Service, The Royal Brisbane and Women’s Hospital, Herston, QLD Australia; 8 Surgical Treatment and Rehabilitation Service (STARS) Education and Research Alliance, The University of Queensland and Metro North Health, Herston, QLD Australia; 9 Physiotherapy Department, Metro North Hospital and Health Service, The Royal Brisbane and Women’s Hospital, Herston, QLD Australia; 10 Centre for Health Research, University of Southern Queensland, Ipswich, QLD Australia

**Keywords:** Musculoskeletal injury, Return to work, Road traffic crash, Interventions, Whiplash, Occupational outcomes

## Abstract

**Background:**

Musculoskeletal injuries are common after road traffic crash (RTC) and can lead to poor work-related outcomes. This review evaluated the impact of interventions on work-related (e.g. sick leave), health, and functional outcomes in individuals with a RTC-related musculoskeletal injury, and explored what factors were associated with work-related outcomes.

**Methods:**

Searches of seven databases were conducted up until 9/03/2023. Eligible interventions included adults with RTC-related musculoskeletal injuries, a comparison group, and a work-related outcome, and were in English. Meta-analyses were conducted using RevMan and meta-regressions in Stata.

**Results:**

Studies (*n* = 27) were predominantly conducted in countries with third-party liability schemes (*n* = 26), by physiotherapists (*n* = 17), and in participants with whiplash injuries (94%). Pooled effects in favour of the intervention group were seen overall (SMD = − 0.14, 95% CI: − 0.29, 0.00), for time to return to work (− 17.84 days, 95% CI: − 24.94, − 10.74), likelihood of returning to full duties vs. partial duties (RR = 1.17, 95% CI: 1.01, 1.36), decreased pain intensity (− 6.17 units, 95% CI: − 11.96, − 0.39, 100-point scale), and neck disability (− 1.77 units, 95% CI: − 3.24, − 0.30, 50-point scale).

**Discussion:**

Interventions after RTC can reduce time to return to work and increase the likelihood of returning to normal duties, but the results for these outcomes were based on a small number of studies with low-quality evidence. Further research is needed to evaluate a broader range of interventions, musculoskeletal injury types, and to include better quality work-related outcomes.

**Supplementary Information:**

The online version contains supplementary material available at 10.1007/s10926-024-10185-z.

## Background

It is estimated that road traffic crash (RTC) injuries will cost the world economy US$1.8 trillion from 2015 to 2030 [1]. In Australia, injuries from RTC were calculated to cost AU$13 billion in 2016 [2], with the main cost being in workplace output losses [3]. Musculoskeletal injuries (e.g. whiplash [4] and fractures [5]) are the most common non-fatal injury from RTC [6]. These injuries often result in persistent pain [7] and poor work outcomes such as sick leave [8], delayed return to work [9], and impaired work ability [10].

Little is known about whether interventions delivered after RTC can shorten time to return to work and improve work outcomes for musculoskeletal injuries. Previous reviews have predominantly been directed at more serious injuries such as traumatic brain injury [11, 12] and spinal cord injury [13, 14], and reviews evaluating musculoskeletal injuries after RTC have not focused on work-related outcomes [15, 16].

The content of interventions, the context in which the interventions are delivered, and concurrent changes in other outcomes have not been explored and could contribute to intervention effectiveness. Components of interventions that may impact intervention effectiveness include intervention length, who delivered the intervention, and their frequency of contact with participants. The research to date on contextual factors suggests that personal and injury-related characteristics such as age, gender, injury type and severity, physical and mental health, job type, return to work expectancies, and socio-economic status [17–21] are important for returning to work after musculoskeletal injury and may also be important to consider in an intervention context. At a workplace and societal level, employer and friend (but not family) support have both been positively associated with returning to work after musculoskeletal injury [17, 21, 22]. At a macro level, differences in compensation schemes within and across countries can impact return to work, such that more supportive compensation schemes can have better return to work rates [23, 24]. Improvements in health and functional outcomes may also be associated with improved work outcomes. Findings from prospective studies report that less that less self-reported pain and higher mental health-related quality of life within three months of a RTC predict higher return to work rates 12–24 months after injury [25, 26].

The primary aim of this systematic review is to evaluate the impact of interventions on work-related outcomes in individuals who have sustained a RTC-related musculoskeletal injury. Secondary aims are to understand the intervention components, participant characteristics, workplace characteristics, and external factors that may be associated with improvement in work-related outcomes in an intervention context (aim 2) and to evaluate the impact of these interventions on health and functional outcomes (aim 3).

## Methods

This systematic review was registered with the PROSPERO (International prospective register of systematic reviews) database on 14/08/2018 (CRD42018103746). The protocol of the review has also been published [27]. The reporting of this review follows the Preferred Reporting Items for Systematic review and Meta-Analysis (PRISMA) 2020 statement [28] (see Supplementary File 1).

### Data Sources and Search Strategy

The electronic databases PubMed, Embase, Web of Science, Cumulative Index to Nursing and Allied Health Literature (CINAHL), PsycINFO, Centre for Controlled Trials (CENTRAL), and ProQuest Dissertations & Theses Global were searched on 17/08/2018, 19/03/2020, and 9/03/2023. Search terms related to four categories: (1) road traffic crash, (2) musculoskeletal injury, (3) work-related outcomes, and (4) intervention design, which were separated by the Boolean phrase ‘AND’. The search strategy for one database can be found in Supplementary File 2. Searches were limited to studies in English. No further limits were used.

### Study Selection

The primary author (CLB) exported the searches to Endnote (Clarivate, London, UK) and removed duplicates. Two authors (CLB and EJS or GW) independently reviewed titles and abstracts using the following inclusion criteria: (1) adults with a musculoskeletal injury of any severity from a RTC, (2) intervention with a comparison group, (3) work-related outcome, and (4) in English. Protocol papers and abstracts were excluded. Study authors were contacted if the cause of the injury was unclear, or if the study reported on a sample with a wide range of injuries, to just extract data on participants with musculoskeletal injuries. For studies that were research protocols or in abstract format only, the primary author sought the full results through additional database searching and then via contacting the study authors for more information. Multiple publications from the one trial were counted as one study. Studies that met the inclusion criteria or required further information were downloaded as full text. Full texts were independently reviewed by the primary author and either EJS, EMG, or GW. The study screening method was updated to Covidence (Veritas Health Innovation Ltd, Melbourne, Australia) for the most recent search, which is an amendment to the review protocol [27].

### Data Extraction

Data from studies that met the inclusion criteria were extracted into tables (independently by author CLB and a research assistant or statistician). Data extracted are outlined in the review protocol [27] and included study information, participant details, work-related outcomes (e.g. days to return to work, amount of sick leave), physical and mental health outcomes, and return to usual activities. Only outcomes reported at the end of the intervention period and any follow-up outcome measurement after this (i.e. intervention maintenance) were extracted. When a percentage was reported instead of the raw numbers, author CLB calculated the number of ‘events’ by multiplying the percent per group with the total number of participants per group. Numbers were rounded up when fractional ≥ 0.5 in accordance with mathematical convention. Study authors were contacted by the primary author for more information or to clarify data if necessary. Characteristics of the studies and participants are presented in Table 1 and intervention characteristics and outcomes are presented in Tables 2 and 3 (summarised) and Supplementary File 3 (in detail). Study quality was measured using the Cochrane risk-of-bias (RoB 2) tool for randomised trials [29] or the Risk of Bias in Non-randomized Studies—of Interventions (ROBINS-I) tool [30] by two authors (CLB and NEA) independently. Discrepancies between the two authors were discussed and resolved through discussion. GRADE was assessed by two authors (CLB and EMG) independently using GRADEpro and discrepancies were resolved through discussion.

**Table 1:** Characteristics of studies and participants

First author, year, country, [reference number]	Comp scheme	N	Injury and condition (injury grade)	% RTC	Time since injury	Age mean (SD)	% women	% employed pre-injury	Compensation status
Ludvigsson, 2017, Sweden [59]; Lo, 2018 [35]	3rd party	216 165^a^	whiplash (II–III) & chronic WAD	80	6–36 months (mean 20 months)	41 (11) years	65	89	20% with unsettled insurance claim
Villafane, 2017, Italy [39]	3rd party	41	whiplash & chronic WAD (I–II)	100	≤ 48 h	41 (11) years	66	NR	NR
Wu, 2017, Australia^b^ [46]	3rd party	214 (84)	RTC-related injuries requiring ≥ 5 days in hospital (musculoskeletal injury)	100	Median 5 days from admission	47 years	32	71^c^	NR
Brooke, 2014, Australia [50]	3rd party	76	fracture injury AIS ≥ 2	100	10 days	42 years	33	87	NR
Conforti, 2013, Italy [36]	3rd party	135	whiplash injury (I–II)	100	Mean 28 days	NR	54	NR	NR
Elbers, 2013, The Netherlands ^b^ [58]	3rd party	176 (42)	RTC-related injuries (whiplash injury)	100	< 2 years (mean 12 months)	49 (15) years	47	78	100% made compensation claim, 41% with settled claim
Lamb, 2013, England, Part 1 [32, 33]	3rd party	3851	acute whiplash & WAD (I–III)	94	≤ 3 days of ED visit	37 (13) years	57	71	~ 64–67% made compensation claim, 38% with settled claim
Lamb, 2013, England, Part 2 [32, 33]	3rd party	599	acute whiplash, WAD (I–III), active symptoms	95	At least 3 weeks past ED visit	40 (13) years	63	73	80% made compensation claim
Schaafsma, 2012, Australia [53]	3rd party	186	non-catastrophic injuries, WAD, soft tissue, joint or orthopaedic	100	1 month	45 years	66	70	100% made compensation claim, ~ 66% with settled claim
Pato, 2010, Switzerland [43]	3rd party	87	whiplash injury (I–II) & persistent neck pain or headache	100	6–12 months (median 10 months)	41 (12) years	62	86	NR^d^
Amirfeyz, 2009, UK [49]	3rd party	141	whiplash injury & WAD (I–III)	100	16 days–3 years (mean 5 months)	40 (14) years	59	89	NR
Ask, 2009, Norway [48]	3rd party	25	whiplash, subacute WAD (I–II)	100	Approximately 6 weeks	37 years	56	NR	NR
Kongsted, 2007, Denmark [42]	3rd party	458	whiplash injury	100	≤ 10 days (median 4 days)	Median age 34 years	72	88	NR
Ottosson, 2007, Sweden [45]	3rd party	127	minor musculoskeletal injuries (ISS < 9)	100	≤ 1 week	43 years	48	68	NR
Vikne, 2007, Norway [44]	3rd party	214	whiplash & chronic WAD (I–II)	100	6–12 months	Age range 18–60 years	67	NR	100% made compensation claim, all unsettled claims
Stewart, 2007, Australia [38]	3rd party	134	whiplash & WAD (I–III)	100	3–12 months (mean symptom duration 9 months)	43 (15) years	66	80	100% made compensation claim, % with settled claim NR
Bunketorp, 2006, Sweden [41]	3rd party	47	whiplash & subacute WAD	98	Mean 64 days	31 years	64	NR	NR
Scholten-Peeters, 2006, The Netherlands [47]	3rd party	80	whiplash & acute WAD (I–II)	100	4 weeks	33 years	66	NR	NR
Sullivan, 2006, Canada [34]	No fault	130	whiplash injury (I–II)	100	NR	41 years	49	100	NR
Ferrari, 2005, Canada [56]	3rd party	112	whiplash injury & WAD (I–II)	100	≤ 72 h	39 years	54	79^e^	51% made compensation claim at 2 weeks, 53% at 3 months
Crawford, 2004, UK [52]	3rd party	108	soft tissue injury of the neck	100	≤ 48 h	34 years	63	77	NR
Ventegodt, 2004, Denmark [54]	3rd party	87	whiplash injury & chronic WAD	100	6 months–10 years (median 37 months)	Median 38 years	83	NR	NR
Rosenfeld, 2003, Sweden [37]	3rd party	97	whiplash injury & WAD (0–II)	100	≤ 96 h	35 years	67	NR	NR
Bonk, 2000, Germany [40]	3rd party	97	acute whiplash & WAD (I–II)	100	≤ 3 days	28 years	46	NR	NR
Borchgrevink, 1998, Norway [55]	3rd party	201	whiplash neck sprain injury	100	At time of ED intake	37 years	60	85	NR^f^
Pettersson, 1998, Sweden [57]	3rd party	40	whiplash injury (II–III)	100	≤ 8 h	35 years	45	75	NR
Provinciali, 1996, Italy [51]	3rd party	60	whiplash injury & cervico-encephalic syndrome	100	≤ 2 months (mean 30 days)	41 years	58	100	NR

*AIS* Abbreviated Injury Scale, *Comp* compensation, *ISS* Injury Severity Score, *WAD* whiplash-associated disorder

^a^Includes only those who were currently employed or employed at time of the accident

^b^Subsample information of participants with musculoskeletal injuries in parentheses

^c^Based on 173 who completed follow-up. Subsample of 84 participants had musculoskeletal injury and were all employed pre-injury

^d^Compensation status not reported but participants recruited through the Swiss Accident Insurance Fund (SUVA) and the Swiss Insurance Association registers

^e^% employed at baseline, prior injury employment not reported

^f^Compensation status not reported but paper reports nearly all participants covered by insurance

**Table 2:** Study design, intervention details, and outcomes of therapeutic interventions

First author, year [reference number]	Study design	Interventionist and intervention/s (length if reported)	Work outcomes reported (timeframe) (Bold outcomes were significantly different between groups [*p* < 0.05])	Health-related and functional outcomes (Bold outcomes were significantly different between groups [*p* < 0.05])
Ludvigsson, 2017 [59]; Lo, 2018 [35]	3-arm RT	Physiotherapist—neck exercise with or without psychological strategies (12 weeks)	Sick leave days (12 months) **Work Ability Index (psychological strategies only) (3, 6, and 12 months)**	Pain (VAS), **NDI**, quality of life (EQ-5D), **EQ-VAS, quality of life (SF-36), PSFS**, HADS
Villafane, 2017 [39]	2-arm non-RCT	Physiotherapist—cognitive behavioural and neck/shoulder exercises, neck collar (15 days)	% on sick leave (2 and 12 weeks)	**Neck pain (VAS), NDI**
Wu, 2017 [46]	2-arm RCT	Increased physiotherapist and/or occupational therapist sessions, weekly case conferences with in-reach rehab team (~ 6 days)	% RTW (4–6 months follow-up) % same job out of those who RTW % full duties out of those who RTW % usual hours out of those who RTW	OMPQ, functional independence, DASS, quality of life (SF-12)
Brooke, 2014 [50]	2-arm non-RCT	Rehabilitation physician—activity limitations consultation (18 weeks)	% RTW (18 weeks) **% return to normal work (18 weeks)**	Pain (VAS), DASS, TSQ, return to driving
Conforti, 2013 [36]	2-arm RCT	Physiotherapist—laser therapy (5 days)	**Days to RTW by end of treatment**	**Pain (VAS)**
Lamb, 2013, Part 2 [32, 33]	2-arm RCT	Physiotherapist—manual therapy, soft tissue techniques, exercise, psychological strategies (8 weeks)	**Workdays lost (4, 8, and 12 months)**	**NDI (at 4 months only)**, quality of life (SF-12), **self-rated benefit (at 4 months only)**
Schaafsma, 2012 [53]	2-arm non-RCT	Enhanced insurance consultation	Employment status (7 months) % returned to full duties (out of those employed at 7-month follow-up)	Pain levels, self-rated recovery, quality of life (SF-12), HADS, **return to usual activities**
Pato, 2010 [43]	3-arm RCT	Physiotherapist—massage, learned relaxation, isometric and isotonic training of neck muscles. Participants were further randomised to receive CBT intervention by psychologist. (8 weeks)	Working capacity (8 weeks, 6 months)	McGill pain questionnaire, Health Assessment Questionnaire, Cognitive Failures Questionnaire, **Well-Being Scale (8 weeks CBT), self-reported recovery (CBT)**
Amirfeyz, 2009 [49]	2-arm non-RCT	Physiotherapist—neck posture advice and practice, graded activities, other exercises (6 weeks)	Prevalence of any sick leave in past 4 weeks (6-week follow-up)	**Neck disability (Bournemouth Questionnaire)**
Ask, 2009 [48]	2-arm RCT	Physiotherapist—motor control training, low loaded training program (6 weeks)	Prevalence of any sick leave (12 months)	Pain (VAS), NDI
Kongsted, 2007 [42]	3-arm RCT	Physiotherapist—active mobilisation with or without neck collar (4–6 weeks)	% of participants with sick days or reduced working hours in past month (12 months), RTW (12 months)	Neck pain intensity, neck disability (Copenhagen Neck Functional Disability Scale), quality of life (SF-36)
Ottosson, 2007 [45]	2-arm RCT	Physiotherapist, anaesthesiologist, psychologist—group sessions on tissue healing, pain management, self-care (4 weeks)	Weeks of sick leave (12 months)	Physical discomfort/pain (VAS), mental distress (VAS), coping capability (VAS), quality of life (SF-36), Short Musculoskeletal Function Assessment, **self-reported recovery**
Vikne, 2007 [44]	4-arm RCT	Physiotherapist—traditional physiotherapy with sling exercises (1 year)	Prevalence of any sick leave (12 months)	Pain (VAS), self-reported disability (Roland and Morris disability score), psychological distress, cervical range of movement
Stewart, 2007 [38]	2-arm RCT	Physiotherapist—graded exercise, CBT components (6 weeks)	% working at follow-up (6 weeks, 12 months), % returned to full duties (6 weeks, 12 months)	**Pain intensity (6 weeks only), NDI (6 weeks only), PSFS (6 weeks only), quality of life (SF-36, 6 weeks only)**
Bunketorp, 2006 [41]	2-arm RCT	Physiotherapist—neck & shoulder exercises, fear of pain & movement, increased self-efficacy for physical activities, individualised, groups of 3–4 later formed (~ 9 weeks)	% with improved sick leave (3 and 9 months)	Pain (VAS, % improved), **Pain Disability Index (3 months only), Tampa Scale for Kinesiophobia (% improved, 3 months only)**, cervical range of motion
Scholten-Peeters, 2006 [47]	2-arm RCT	Physiotherapist—education, advice, graded activity, exercise therapy (~ 20 weeks)	Work activities in daily living (12 months)	Neck pain (VAS), NDI, quality of life (SF-36), **% functionally recovered (higher in comparison group)**, cervical range of motion, improvement in coping strategy
Sullivan, 2006 [34]	2-arm non-RCT	Physical therapists and occupational therapists—Progressive Goal Attainment Program: activity monitoring & prescription, graded activity, cognitive restructuring (10 weeks)	**% RTW (14 weeks)**	McGill Pain Questionnaire, Pain rating index, Pain Disability Index, **Pain Catastrophizing Scale**, Tampa Scale for Kinesiophobia
Crawford, 2004 [52]	2-arm RCT	Advice sheet with mobilisation exercise regime, told to stop using soft collar (12 months)	**Days to RTW (12 months)**	Pain (VAS), range of movement
Ventegodt, 2004 [54]	2-arm RCT	Teachings in philosophy of life, gestalt psychotherapy and body therapy (2 months)	Sick leave (3 months)	Neck pain intensity, arm pain intensity, quality of life (global)
Rosenfeld, 2003 [37]	4-arm RCT	Physiotherapist—postural control and cervical rotation exercises with 96 h or after 14 days of injury (~ 6 weeks)	Sick leave days (6 months post baseline), **Sick leave days (3 years post baseline)**	**Pain intensity (6 months and 3 years)**, cervical range of movement
Bonk, 2000 [40]	2-arm RCT	Physiotherapist—mobilisation of the neck, strength and isometric exercises, advice to avoid collar (3 weeks)	% with any work missed	**Neck pain (6 weeks not 12 weeks), shoulder pain (6 weeks not 12 weeks)**, arm pain, range of motion
Provinciali, 1996 [51]	2-arm RCT	Relaxation training, postural training, manual treatment of the spine, psychological support, eye exercises, soft collar (2 weeks)	**Days to return to work (6 months)** In usual occupation (6 months)	**Pain intensity**, range of movement

*CBT* cognitive behaviour therapy, *DASS* The Depression, Anxiety and Stress Scale, *HADS* The Hospital Anxiety and Depression Scale, *NDI* Neck Disability Index, *OMPQ* Örebro Musculoskeletal Pain Questionnaire, *PSFS* Patient-Specific Functional Scale, *RCT* randomised controlled trial, *RTW* return to work, *SF* Short Form, *TSQ* Trauma Screening Questionnaire, *VAS* visual analogue scale, *WAD* whiplash-associated disorder

**Table 3:** Study design, intervention details, and outcomes of emergency department, drug-based, and web-based interventions

First author, year, [reference number]	Study design	Interventionist and intervention/s	Work outcomes reported (timeframe) (Bold outcomes were significantly different between groups [*p* < 0.05])	Health-related and functional outcomes (Bold outcomes were significantly different between groups [*p* < 0.05])
Emergency department (ED) interventions				
Lamb, 2013, Part 1 [32, 33]	2-arm cluster-RCT	ED clinician—one-off consultation: reassurance, encouraging return to normal activities and neck exercises, avoidance of neck collar + ‘The Whiplash Book’	Workdays lost (4, 8, 12 months)	NDI, quality of life (SF-12), **self-rated benefit (at 12 months only)**
Ferrari, 2005 [56]	2-arm RCT	Research nurse—one-off 1-page whiplash pamphlet summarised from the Whiplash Book	Employed (3-month follow-up) % off work (3-month follow-up) % with any missing workdays (3-month follow-up)	Any pain, neck pain, shoulder pain, low back pain, self-reported recovery
Borchgrevink, 1998 [55]	2-arm RCT	ED clinician—one-off act as usual advice, instructions in self-training of the neck	% with any sick leave post 14 days (6 months) % on 100% sick leave (6 months) % on 50% sick leave (6 months)	**Pain (VAS)**, subjective feeling of global improvement
Drug-based interventions				
Pato, 2010 [43]	3-arm RCT	Physician—bupivacaine injection into tender point, 2 x week, 8 weeks Physician—1 × 200 mg flurbiprofen/day	Working capacity (8 weeks, 6 months)	McGill pain questionnaire, Health Assessment Questionnaire, Cognitive Failures Questionnaire, Well-Being Scale, self-reported recovery
Pettersson, 1998 [57]	2-arm RCT	Hospital pharmacist—one-off treatment high-dose methylprednisolone administered within 8 h after injury	**Sick days (6 months)** **% on sick leave due to WAD (6 months)** **Sick leave profile (6 months)**	Neck pain, arm pain, depression symptoms, concentration problems, memory impairment, anxiety symptoms
Web-based interventions				
Elbers, 2013 [58]	2-arm RCT	Website with information about compensation process (49 pages), 5-lesson problem-solving therapy, 10 frequently asked questions	3-item Work Ability Index (3, 6, 12 months)	EQ-VAS, SCL-90 (depression and anxiety subscales)

*NDI* Neck Disability Index, *RCT* randomised controlled trial, *SCL-90* The Symptom Checklist-90, *SF* Short Form, *VAS* visual analogue scale, *WAD* whiplash-associated disorder

### Data Analysis

To address aims 1 and 3, meta-analyses were performed in RevMan (version 5.4; Cochrane, London, UK) for the work, health, and functional outcomes provided that the same outcome was reported in at least three studies. Studies which had three or more arms were condensed into two arms for the meta-analysis as per Cochrane’s formulae for combining groups in Chap. 6 of the Cochrane Handbook [31]. If the study arms were considered too different to combine, the control group numbers were divided by the number of intervention groups to provide a comparison for each intervention arm. Original units were used for continuous outcomes and risk ratios were calculated for the categorical outcomes. The standardised mean difference (SMD) and standard error were also calculated for work-related outcomes either as Hedge’s g for continuous outcomes or converting odds ratios as per the Cochrane Handbook guidance for categorical outcomes (Chap. 10 [31]). Publication bias was evaluated using funnel plots created in Stata (version 17; StataCorp, College Station, Texas, USA). Study heterogeneity was reported using Tau-squared and I-squared statistics. Subgroup analyses were conducted for each work outcome to compare interventions versus usual care/control and intervention versus interventions if there were at least two studies in each subgroup. Subgroup analyses including only studies with a significant work outcome were also performed for the health and functional outcomes when there were at least two studies. Sensitivity tests using the ‘leave-one-out’ approach were used to explore if the results were reliant on any one study.

To address aim 2, predictors of work outcomes at the individual study level were extracted from papers when reported and described narratively. Meta-regressions were conducted in Stata (version 17).

## Results

**Fig. 1:**
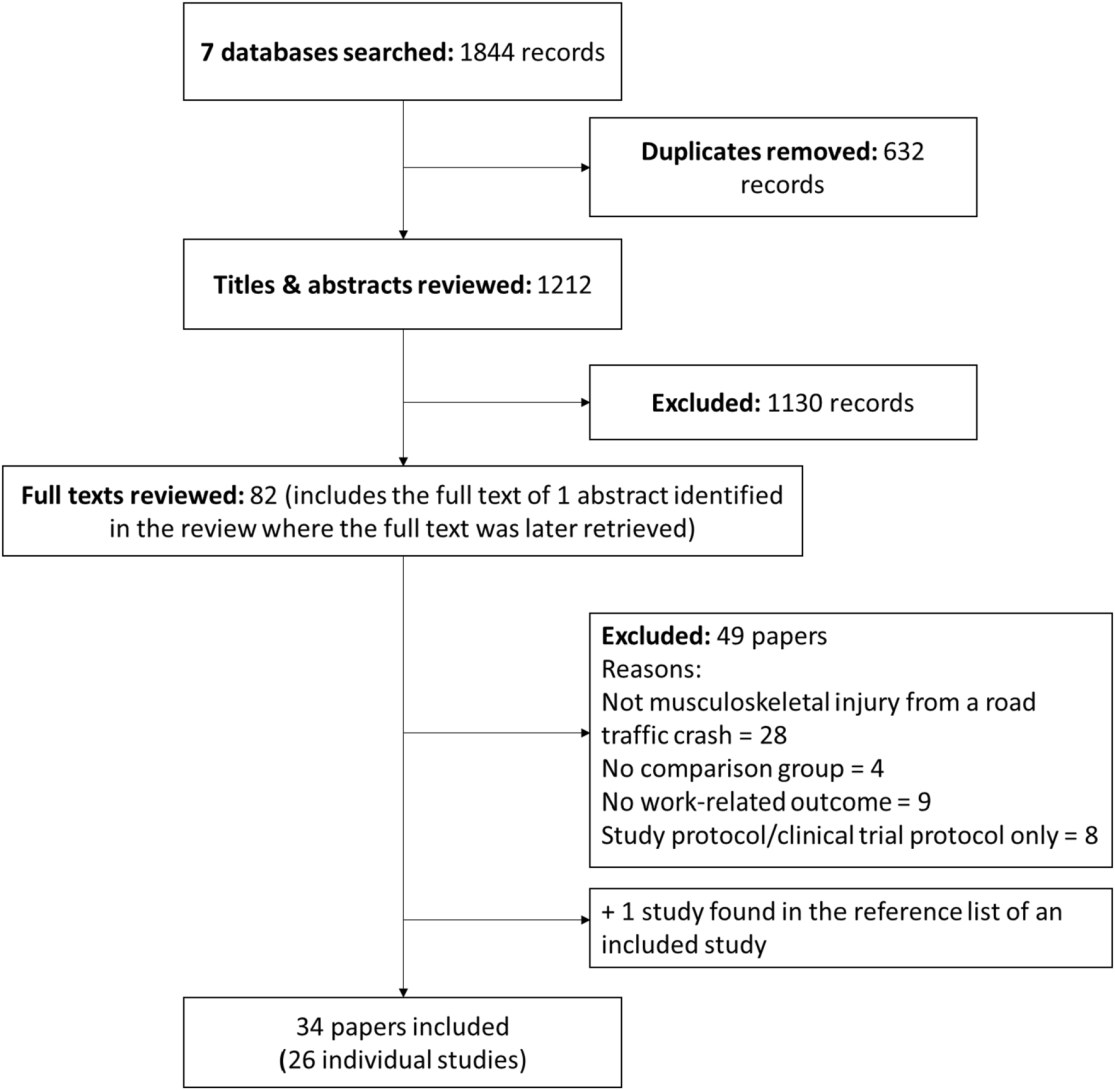
Study flow chart

Following exclusion of 632 duplicates, the searches identified 1212 records from the seven databases (see Fig. 1). One additional paper was found by searching the reference lists of included studies. In total, 34 papers met inclusion criteria, for a total of 26 individual studies. One study [32, 33] included a two-part trial, an initial cluster-RCT evaluating one intervention, and then a nested RCT evaluating a different intervention. For ease of reporting, this trial will be reported as two separate studies from here on, for a total of 27 studies. Studies that were reviewed in full text, but did not meet inclusion criteria, are listed in Supplementary File 4.

### Study Details and Compensation Schemes

Studies were published between 1996 and 2018, see Table 1. The total number of participants across the studies was 7571 (ranging from 25 to 3851 participants). Studies were predominantly from Europe (*n* = 21), two studies were from Canada, and four from Australia (New South Wales only). Most studies (*n* = 22) were randomised trials of two to four arms; five studies were non-randomised. At the time that the studies were conducted, almost all except one [34] were conducted in countries that had a third-party liability scheme. The percentage of participants who had pursued an insurance claim varied between 53 and 100% across studies.

### Participant Characteristics

Mean ages of participants ranged from 27 to 48 years. On average, samples had slightly more women than men (58% women, range 32 to 83%). The majority (94%) of participants had whiplash injuries (7093 participants, 24 studies). Other injuries included musculoskeletal, orthopaedic, or fracture injuries (e.g. upper and lower limb injuries; 262 participants, 3 studies), soft tissue injuries (143 participants, 2 studies), contusions (13 participants, 1 study), and joint injuries (9 participants, 1 study). Time since injury (or emergency department intake) for participants was within 10 days in 12 studies, between 3 weeks and 2 months in 7 studies, within 3 years in 6 studies, and within 10 years in 1 study.

### Intervention Types, Comparison Groups, and Effectiveness

#### Therapeutic Interventions

Twenty-two studies (out of 27, 81%) evaluated one or more therapeutic intervention/s, see Table 2. Physiotherapists were the most common therapeutic interventionists (17 studies, 77%). Only four physiotherapy interventions (out of 17, 24%) had a significant work-related difference post intervention compared to a comparison group. Three of these interventions consisted of both graded exercise and psychological strategies for 8–12 weeks compared to usual care or minimal intervention [32, 34, 35]. The fourth intervention evaluated daily laser therapy for five days compared to conventional physiotherapy [36]. A 6-week cervical rotation intervention (compared to advice leaflet) had no significant work-related outcome post intervention (6 months) but did at 3-year follow-up [37]. Cognitive behavioural exercise interventions of shorter lengths (2 or 6 weeks [38, 39]), neck mobilisation or neck training programs [40–43], sling exercises [44], group sessions [45], and increases in therapist visits in hospital [46] were not effective on work outcomes. Physiotherapy intervention compared to general practice [47], motor control training compared to neck exercises [48], and neck posture advice received after injury compared to three months post injury [49] did not have any intervention effect on work outcomes.

Other therapeutic interventions that were effective on work-related outcomes were consultations with a rehabilitation physician compared to usual care [50], a 2-week multi-component postural and psychological intervention compared to physical agents [51], and an early mobilisation exercise regime advice sheet compared to a soft collar [52]. Enhanced insurance consultation [53], Cognitive Behavioural Therapy [43], and philosophy of life training [54] did not have significant effects on work-related outcomes.

### Interventions Delivered in the Emergency Department

Interventions delivered in the emergency department (ED) were not effective at improving work-related outcomes compared to soft collar use or usual care, see Table 3. Interventions were ‘act as usual’ advice given by an ED clinician [55], provision of the ‘Whiplash Book’ and an active management consultation delivered by an ED clinician [32], and a 1-page pamphlet summary of the Whiplash Booklet [56].

### Drug-based Interventions

Two studies evaluated the impact of medication on work outcomes in participants with whiplash injury [43, 57] (see Table 3). High-dose methylprednisolone administered within 8 h of injury resulted in improved sick leave outcomes after 6 months compared to placebo treatment [57]. Twice-weekly bupivacaine injection for 8 weeks improved physician-determined working capacity over time, but was not significantly better at improving working capacity versus daily flurbiprofen (200 mg) tablets or twice-weekly physiotherapy [43].

### Web-Based Interventions

A website with information about the compensation process and five lessons on problem-solving therapy was not significantly better at improving self-reported work ability after 12 months compared to a control website with links to existing information [58] (see Table 3).

### Work-Based Interventions

No studies evaluated the impact of a work-based intervention (e.g. job redesign or adaptation of working hours) on work-related outcomes.

### Work-Related Outcomes and Meta-analyses

#### Days to Return to Work

**Fig. 2:**
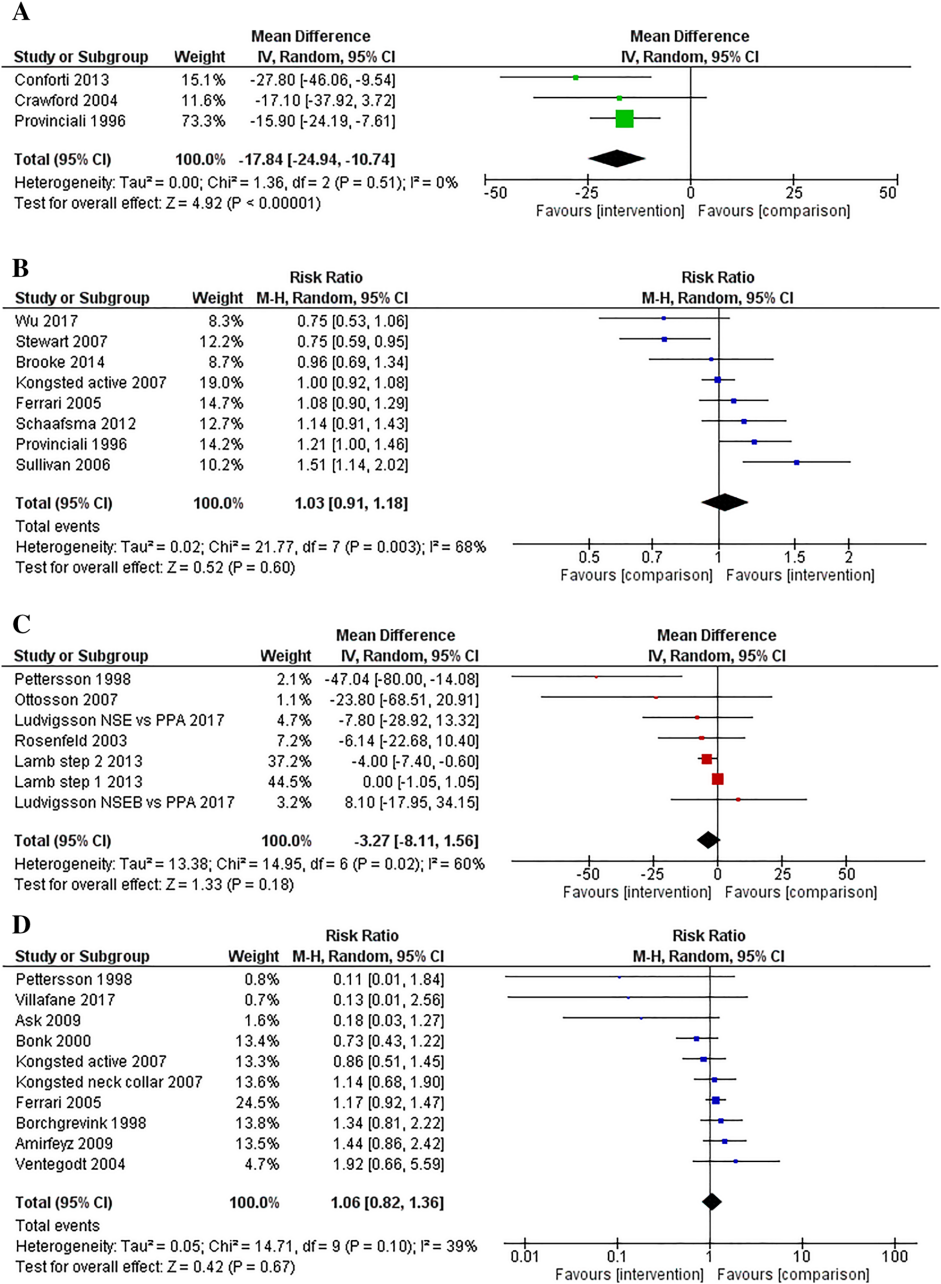

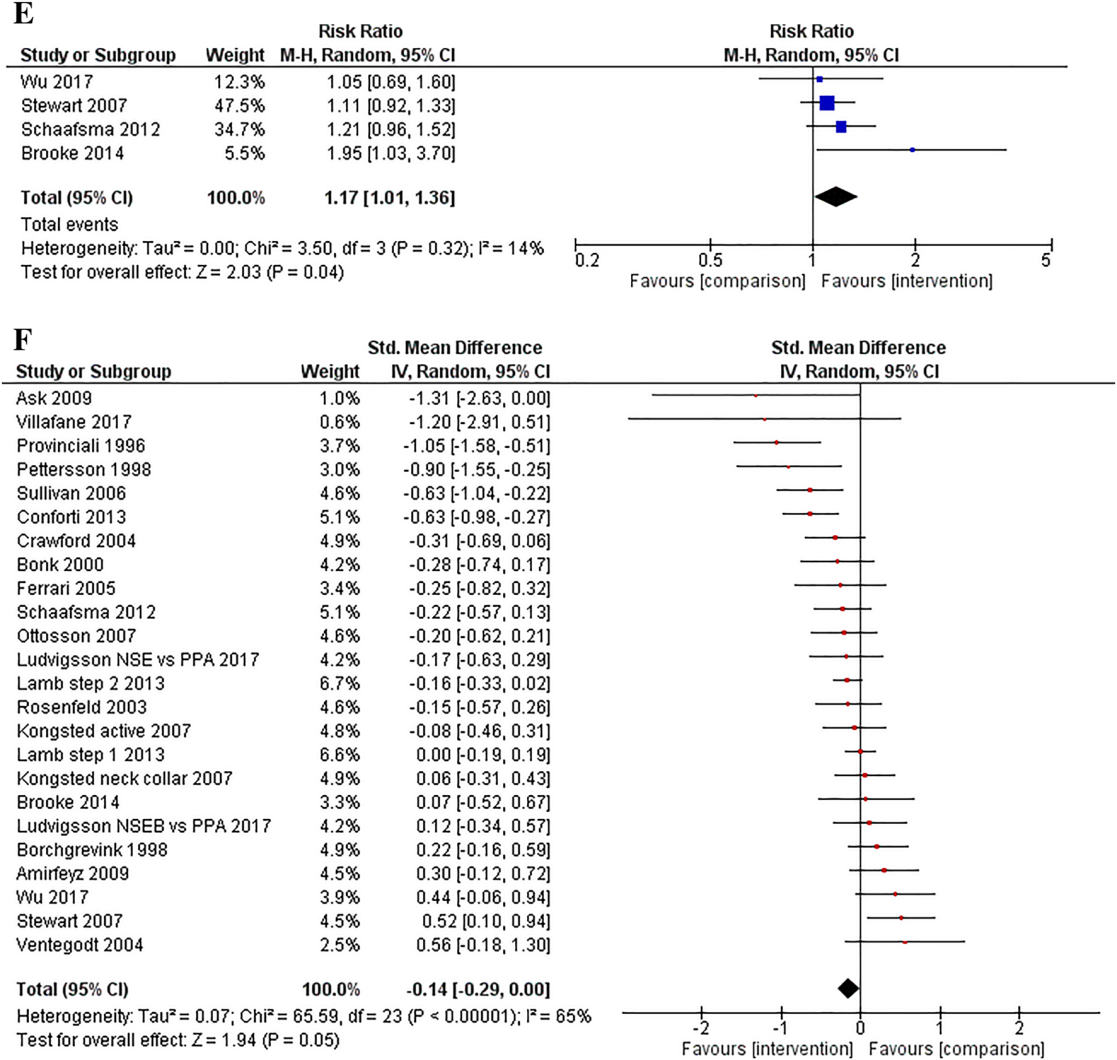
Forest plots for the meta-analyses: days to return to work (**2A**), percentage of participants who returned to work or were employed at follow-up (**2B**), days of sick leave (**2C**), percentage of participants with sick leave (**2D**), percentage of participants returning to full or normal duties out of those who had returned to work (**2E**), and standardised effects for return to work and sick leave outcomes (**2F**)

Interventions were effective compared to a comparison group for improving days to return to work (3 studies, − 17.84 days, 95% CI: − 24.94, − 10.74, *p* < 0.001; SMD = − 0.62, 95% CI: − 1.00, − 0.24; see Fig. 2A). No publication bias was evident when viewing the funnel plot and there was no heterogeneity (Tau^2^ = 0.00, *I*^2^ = 0%).

### Percentage of Participants Returned to Work or Employed at Follow-Up

Interventions were not effective compared to a comparison group for percentage of participants returned to work or being employed at follow-up (8 studies, risk ratio = 1.03, 95% CI: 0.91, 1.18, *p* = 0.60; see Fig. 2B). No publication bias was evident but there was significant (*p* = 0.003) heterogeneity (Tau^2^ = 0.02, *I*^2^ = 68%). Subgroup analyses comparing interventions versus usual care/control and intervention versus interventions found no significant intervention effects (see Supplementary File 5).

### Days of Sick Leave

Interventions were not effective compared to a comparison group for decreasing days of sick leave (6 studies, 7 comparisons, − 3.27 days, 95% CI: − 8.11, 1.56, *p* = 0.18; SMD = − 0.12, 95% CI: − 0.26, 0.03; see Fig. 2C). There was significant (*p* = 0.02) heterogeneity (Tau^2^ = 13.38, *I*^2^ = 60%) and the funnel plot revealed some asymmetry as the smaller, less precise studies reported larger effects in favour of the intervention group than the more precise studies. When these studies were removed, heterogeneity was improved (Tau^2^ = 3.35, I^2^ = 36%). There was a significant intervention effect (− 3.98 days, 95% CI: − 7.25, − 0.72, *p* = 0.02) when evaluating only studies that compared intervention to intervention, with low heterogeneity (Tau^2^ = 0.00, *I*^2^ = 0%) (see Supplementary File 5). When comparing intervention to a usual care or control group, the effect was large (− 20.35 days, 95% CI: − 53.30, 12.60) but not significant (*p* = 0.23) and with high heterogeneity (Tau^2^ = 632.91, *I*^2^ = 78%).

### Percentage of Participants with Sick Leave

Interventions were not effective compared to a comparison group for amount of sick leave (any sick leave during the study or being on sick leave at follow-up) (9 studies, 10 comparisons, risk ratio = 1.06, 95% CI: 0.82, 1.36, *p* = 0.67; see Fig. 2D). There was low heterogeneity (Tau^2^ = 0.05, *I*^2^ = 39%); however, the funnel plot revealed the same asymmetry as for days of sick leave. Effects did not change substantially when interchanging different sick leave outcomes (e.g. when studies reported both ‘any sick leave post injury’ and ‘on sick leave at follow-up’). Subgroup analyses comparing interventions versus usual care/control and intervention versus interventions found no significant intervention effects.

### Other Work Outcomes

Out of those participants who returned to work, there was a significant pooled effect for returning to full or normal duties at follow-up (4 studies, risk ratio = 1.17, 95% CI: 1.01, 1.36, *p* = 0.04; see Fig. 2E), with no evidence of publication bias and low heterogeneity. Other work-related outcomes—a 3-item measure of the Work Ability Index [58], working capacity [43], and work activities [47]—found no significant intervention effects. One study found a significant intervention effect for the 7-item Work Ability Index [35], but not on days of sick leave [59].

### Standardised Effects

Twenty-two studies (24 comparisons) with standardised return to work, employment, or sick leave outcomes were included in the overall meta-analysis, see Fig. 2F. There was a small effect supporting the intervention groups (SMD = − 0.14, 95% CI: − 0.29, 0.00; *p* = 0.05). There was however significant heterogeneity (Tau^2^ = 0.07, *I*^2^ = 65%, *p* < 0.001). The funnel plot did not show publication bias, and this was supported by results on the Egger test finding no small-study effects (*z* = − 1.54, *p* = 0.12). Subgroup analyses comparing interventions versus usual care/control and intervention versus interventions found no significant intervention effects.

#### Sensitivity Analyses

The leave-one-out sensitivity analyses revealed that the effects for any work outcome were not reliant on any one trial (see Supplementary File 6).

### Characteristics Associated with Work-Related Outcomes

Three studies reported on predictors of work outcomes at the individual study level. Better work outcomes were significantly predicted by a shorter absence from work and greater reductions in pain catastrophising [34]. Worse work outcomes were significantly associated with moderate to heavy loads on the neck [35], a poorer financial situation [35], higher baseline levels of depression and pain-related disability [35], and lawyer involvement [43]. Meta-regression results using the standardised effect scores identified no associations between 17 participant, intervention, external or measurement characteristics and work outcomes (see Supplementary File 7).

#### Overall Effects on Health- and Functional-Related Outcomes, and in Studies with Significant Work Outcomes

Common health- and functional-related outcomes that could be included in the meta-analyses were pain intensity (general pain or neck pain) measured by a visual analogue scale (14 studies), the Neck Disability Index (NDI, 7 studies), self-reported recovery (7 studies), physical and mental health-related quality of life subscales of the Short-Form 12 and 36 (6 studies), and prevalence of neck pain (3 studies). There were significant pooled intervention effects for pain intensity (− 6.17 units, 95% CI: − 11.96, − 0.39, 100-point scale) and the NDI (− 1.77 units, 95% CI: − 3.24, − 0.30, 50-point scale) (see Supplementary File 8), all other pooled effects were not significant. In the studies that had a significant work outcome, only the NDI showed a significant pooled intervention effect (− 2.01 units, 95% CI: − 3.02, − 0.99), the effect for pain intensity was no longer significant.

The following health- and functional-related outcomes were considered too different or there were insufficient studies to combine into meta-analyses. Measures of physical functioning were the Patient-Specific Functional Scale (2 studies, both with significant intervention effects) and cervical/neck range of motion (7 studies, none with significant intervention effects). Psychological functioning outcomes included depression and anxiety (6 studies, none with significant intervention effects), kinesiophobia (3 studies, 1 study with a significant intervention effect), pain catastrophising (1 study with a significant intervention effect), and self-reported well-being (1 study with a significant intervention effect) (see Supplementary File 8).

#### Study Quality

Seventeen randomised studies were classified as being ‘high risk’ and five were classified as having ‘some concerns’ (see Supplementary File 9). No studies were classified as being ‘low risk’. Key issues were missing outcome data, deviations from intended interventions, and selection of the reported result. All five non-randomised studies received an overall risk-of-bias judgement of ‘serious’. In most cases, this was because studies did not control for possible confounding.

#### GRADE and Recommendations

The overall GRADE assessment results for the work-related outcomes were classed as very low (see Supplementary File 10); as such, caution should be taken when interpreting these results. The GRADE assessments were downgraded due to the poor study quality mentioned above, the inconsistency around the estimate for ‘percentage of participants returned to work or employed’ and ‘days of sick leave’ due to high heterogeneity, the imprecision around most of the estimates due to the small overall sample size, and the possibility of publication bias for the two sick leave outcomes.

## Discussion

The aims of this systematic review were to evaluate the impact of interventions on work-related outcomes after traffic crash-related musculoskeletal injury; to explore intervention components, participant characteristics, and external factors; and health and functional outcomes. Meta-analyses with significant intervention effects were days to return to work (~ 18 days difference) and return to full or normal duties (RR = 1.17), supporting the effects of interventions on these outcomes. Across work outcomes, there was a small non-significant effect (SMD = − 0.14, *p* = 0.05) supporting the interventions. Interventions that included both physiotherapy and a psychological component appeared promising for work-related outcomes, whereas ‘one-off’ advice or information interventions delivered in the ED were less promising. Surprisingly, this review found no studies that evaluated specific workplace-based interventions, suggesting more research is needed in this area for this population. Only three studies specifically evaluated predictors of work-related outcomes in their individual studies, identifying that lawyer involvement and personal, injury-related, and psychological factors predict work outcomes in intervention contexts. Workers with these characteristics may be at risk of having difficulties returning to work and may need additional interventional or structural support over and above planned interventions. There is a need for more intervention studies to evaluate predictors of work-related outcomes, to better tailor what individual or intervention-related factors should be addressed. Across studies, there did not seem to be any participant, intervention, or external characteristics that were associated with intervention effects on work-related outcomes. Pain intensity was the most reported non-work outcome and was significantly reduced across studies (average pain decrease of 6 out of 100), but was not significant when just evaluating studies with significant work outcomes. The Neck Disability Index was statistically significantly reduced across studies, including in studies with a significant work outcome (average decrease of 2 out of 50). Overall, intervention effects for work outcomes appeared to occur independently of changes in health- and functional-related outcomes.

Graded exercise and psychological strategies had a promising effect on work outcomes. Three successful physiotherapy interventions consisted of both graded exercise and other psychological strategies delivered between 8- and 12-week duration [32, 34, 35]. These psychological strategies included cognitive behavioural approaches (e.g. goal setting, cognitive restructuring) [33–35] and relaxation techniques [35]. In addition, a short, 2-week intervention of relaxation training, postural training, and psychological support also had a significant intervention effect on work-related outcomes [51]. Psychological strategies may be a necessary component of return to work interventions. Other research in musculoskeletal disorders and mental disorders has found that psychological interventions can improve work-related outcomes [60]. Furthermore, one study in this review [34] identified that reductions in pain catastrophising (a target of the intervention) were associated with a higher rate of return to work.

There is also growing support for interventions to include both exercise and psychological strategies for a range of beneficial outcomes. For example, there is meta-analytic evidence that the combination of both physiotherapy and psychological strategies (e.g. cognitive behavioural therapy) is more effective for physical function outcomes than physiotherapy alone [61]. The combination of both exercise and psychology strategies has also been found to be beneficial for other outcomes such as stress, depression symptoms, perceived recovery, and pain compared to exercise alone in a randomised trial [62]. Notably, some interventions in this review using psychological components did not affect work outcomes, but this may have been a result of the time frame being too short to have an effect [38, 39] or having an active comparison group [43].

Four studies evaluated neck mobilisation or neck training programs, and three studies delivered advice or a pamphlet in the emergency department, with none of these studies finding a significant effect on work-related outcomes. These findings are consistent with other reviews that found that biofeedback interventions of the neck had no effect on work ability [63] and educational interventions to be ineffective for neck pain [64]. The remaining interventions were variable and were rarely evaluated across more than one study.

A pattern that emerged across the studies was the presence of significant findings for a continuous measure of work outcomes (e.g. days to return to work) but not for categorical measures. This suggests that interventions may be less likely to detect intervention effects when only measuring work-related outcomes categorically (i.e. returned to work yes or no). Continuous measures of work-related outcomes may provide a more accurate indication of intervention effects and are important to include in future intervention studies after musculoskeletal injury, a finding also noted in another systematic review [60]. These findings could be used to inform future development of core work outcome measures for whiplash-associated disorder and other musculoskeletal injuries [65]. Another consideration is that we did not have access to all the measures collected by these studies. It is possible that some studies that reported a categorical work-related outcome may have also collected continuous work-related outcomes but did not report them if they were not significant. Another point to note is that the work-related outcome was rarely the primary outcome in the studies. Only six studies listed their work-related outcome as primary or had the work-related outcome as part of a list of outcomes to determine the efficacy of the intervention with no primary outcome listed. Only one study was adequately powered to detect intervention effects on their work-related outcome [42]. In addition, other work-related outcomes besides return to work and sick leave were rarely measured, and no studies reported on job performance or presenteeism. These findings speak to the need to include measures of work outcomes outside the most common ones identified in this review.

The studies included in this review showed overall significant improvements in pain intensity and the Neck Disability Index. This is consistent with other reviews [66, 67] showing the effectiveness of therapeutic and pharmacological interventions on pain and disability outcomes after musculoskeletal injury. However, a significant improvement in pain intensity or neck disability did not always occur in parallel with improved work outcomes. Six studies with either improvements in pain or neck disability showed positive work improvements [32, 35–37, 40, 51], whereas five studies with pain or disability improvements showed no improvement in work outcomes [38, 39, 41, 49, 55]. There was also limited consistency in the other functional and mental health outcomes reported. Similar inconsistencies between outcomes were identified in a review by Finnes et al. (2019) evaluating mental health and sickness absence [60]. Overall, improving mental and physical health on their own may not be sufficient for successful return to work, and work strategies should be specifically targeted.

This review has strengths and limitations. The studies in this review were generally of low quality due to how missing outcome data were dealt with, deviations from intended interventions, and how results were reported. Hence, we have low certainty about our results. The systematic review only included papers published in English and published in peer-reviewed journals. As such, we may have missed studies published in other languages or published in other methods, for example government reports. The studies identified were also primarily limited to fault-based schemes and as such, the findings are not generalisable to interventions delivered under no-fault schemes. It is possible that the interventions evaluated may be more effective under a no-fault scheme, given the negative impacts that compensation stress can have on recovery [68]. Our analysis of days to return to work is potentially biased as it excludes participants who did not return to work. Across the three studies, one study reported that 1 participant did not return to work [52], one study reported that 6 participants in the control group and 1 participant in the intervention group did not return to work [51], and one study did not report if there were any participants who did not return to work [36]. If we had access to the participant-level data, then a hazard ratio analysis would have been more appropriate as it would take into account these missing participants.

A strength of this review is how work outcomes have been separated into different types based on whether they were categorical or continuous, and whether they related to return to work, sick leave, or full/partial duties. Other reviews evaluating work outcomes post injury have focused primarily on categorical return to work (yes/no) or employed (yes/no) outcomes and have not explored the potential impact of how these work outcomes have been measured [11–13, 65]. Further research could explore whether different interventions have an impact on different work outcomes. For example, interventions that target work readiness may reduce time to return to work and interventions that target pain reduction may be more relevant for the amount of ongoing sick leave. Another strength of this review is how it evaluated the multilevel factors that can impact on work outcomes. Many reviews are focused mostly on outcomes from interventions, rather than the contextual factors that are important as well.

In conclusion, interventions delivered to those with musculoskeletal injuries after road traffic crashes have some effectiveness on work-related outcomes. The significant improvements were seen in days to return to work and return to normal or full work duties; however, these findings were based on only three or four studies and the quality of evidence is very low. The evidence suggests that further work should be done to evaluate work-based interventions, to evaluate musculoskeletal injuries from road traffic crash broader than whiplash injury, to include more continuous measures of work-related outcomes and outcomes in addition to sick leave and return to work, and to improve the methodologically quality of the research, which to date is predominantly of low quality.

## Supplementary Information

Below is the link to the electronic supplementary material.

Supplementary material 1 (DOCX 33.7 kb)

Supplementary material 2 (DOCX 15.7 kb)

Supplementary material 3 (DOCX 62 kb)

Supplementary material 4 (DOCX 18.0 kb)

Supplementary material 5 (DOCX 16.2 kb)

Supplementary material 6 (DOCX 23.2 kb)

Supplementary material 7 (DOCX 22.2 kb)

Supplementary material 8 (DOCX 18.8 kb)

Supplementary material 9 (DOCX 146.8 kb)

Supplementary material 10 (DOCX 19.7 kb)

## Data Availability

Extracted data are presented across tables and figures in this manuscript and supplementary files. Additional data can be requested from the primary author.
