# Supplementary material for: Effectiveness of Interventions on Work Outcomes After Road Traffic Crash-Related Musculoskeletal Injuries: A Systematic Review and Meta-analysis
Source: J Occup Rehabil. 2024 Apr 5;35(1):30–47. doi: 10.1007/s10926-024-10185-z (PMC11839784; doi:10.1007/s10926-024-10185-z)
Supplement: Supplementary file 2 — Supplementary material 2 (DOCX 15.7 kb) [file 10926_2024_10185_MOESM2_ESM.docx]

Supplementary File 2. Search Terms

PubMed search

| Work-related terms | work[Title/abstract] OR job[Title/abstract] OR jobs[Title/abstract] OR occupation[Title/abstract] OR occupations[Title/abstract] OR occupational[Title/abstract] OR vocation[Title/abstract] OR vocations[Title/abstract] OR vocational[Title/abstract] OR employee[Title/abstract] OR employees[Title/abstract] OR employer[Title/abstract] OR employers[Title/abstract] OR worker[Title/abstract] OR workers[Title/abstract] OR workplace[Title/abstract] OR employability[Title/abstract] OR unemployment[Title/abstract] OR employment[Title/abstract] OR absentee[Title/abstract] OR “sick listed”[Title/abstract] OR sicklisted[Title/abstract] OR “sick leave”[Title/abstract] OR “sick absence”[Title/abstract] OR “sickness leave”[Title/abstract] OR “sickness absence”[Title/abstract] OR “sick days”[Title/abstract] OR “sick day”[Title/abstract] OR absenteeism[Title/abstract] OR presenteeism[Title/abstract] OR “workday loss”[Title/abstract] OR “workdays lost”[Title/abstract] OR workloss[Title/abstract] OR “disability evaluation”[Title/abstract] OR “disability prevention”[Title/abstract] OR “functional capacity evaluation”[Title/abstract] OR productivity[Title/abstract] OR “disability leave”[Title/abstract] OR “medical leave”[Title/abstract] OR “illness day” [Title/abstract] OR “illness days” [Title/abstract] OR work[MeSH] OR employment[MeSH] OR “Rehabilitation, Vocational”[MeSH] OR “sick leave”[MeSH] OR “work capacity evaluation”[MeSH] |
| --- | --- |

| RTC-related terms | ((car[Title/abstract] OR cars[Title/abstract] OR truck[Title/abstract] OR trucks[Title/abstract] OR automobile[Title/abstract] OR automobiles[Title/abstract] OR vehicle[Title/abstract] OR vehicles[Title/abstract] OR vehicular[Title/abstract] OR cycle[Title/abstract] OR cycles[Title/abstract] OR cyclist[Title/abstract] OR cyclists[Title/abstract] OR cycling[Title/abstract] OR bicycle[Title/abstract] OR bicycles[Title/abstract] OR pedestrian[Title/abstract] OR pedestrians[Title/abstract] OR passenger[Title/abstract] OR passengers[Title/abstract] OR driver[Title/abstract] OR drivers[Title/abstract] OR motor[Title/abstract] OR motorbike*[Title/abstract] OR motorcar[Title/abstract] OR motorcars[Title/abstract] OR motorcycl*[Title/abstract] OR motorist[Title/abstract] OR motorists[Title/abstract] OR motorvehicle[Title/abstract] OR motorvehicles[Title/abstract] OR transport[Title/abstract] OR transportation[Title/abstract] OR traffic[Title/abstract] OR road[Title/abstract] OR roads[Title/abstract] OR roadside[Title/abstract] OR roadsides[Title/abstract]) AND  (accident[Title/abstract] OR accidents[Title/abstract] OR collision[Title/abstract] OR collisions[Title/abstract] OR crash[Title/abstract] OR crashes[Title/abstract] OR crashed[Title/abstract] OR smash[Title/abstract] OR smashes[Title/abstract] OR smashed[Title/abstract])) OR ((road[Title/abstract] OR traffic[Title/abstract]) AND (injury[Title/abstract] OR injuries[Title/abstract] OR trauma[Title/abstract])) OR whiplash[Title/abstract] OR “Whiplash injuries”[MeSH] OR “Accidents, Traffic”[MeSH] |
| --- | --- |

| Musculoskeletal injury | ((ankle[Title/abstract] OR ankles[Title/abstract] OR “anterior cruciate ligament” [Title/abstract] OR arm[Title/abstract] OR arms[Title/abstract] OR back[Title/abstract] OR cervical[Title/abstract] OR elbow[Title/abstract] OR elbows[Title/abstract] OR femur[Title/abstract] OR femoral[Title/abstract] OR fibula[Title/abstract] OR fibular[Title/abstract] OR finger[Title/abstract] OR fingers[Title/abstract] OR foot[Title/abstract] OR feet[Title/abstract] OR forearm[Title/abstract] OR forearms[Title/abstract] OR hand[Title/abstract] OR hands[Title/abstract]  OR hip[Title/abstract] OR hips[Title/abstract] OR humerus[Title/abstract] OR humeral[Title/abstract] OR knee[Title/abstract] OR knees[Title/abstract] OR leg[Title/abstract] OR legs[Title/abstract]  OR ligament[Title/abstract] OR “low back”[Title/abstract] OR “lower back”[Title/abstract] OR “lower limb”[Title/abstract] OR “lower limbs”[Title/abstract] OR “lower extremity”[Title/abstract] OR “lower extremities”[Title/abstract] OR lumbar[Title/abstract] OR mandibular[Title/abstract]OR maxillofacial[Title/abstract] OR metatarsal[Title/abstract] OR metatarsals[Title/abstract] OR muscle[Title/abstract] OR musculoskeletal[Title/abstract] OR neck[Title/abstract] OR pelvis[Title/abstract] OR pelvic[Title/abstract] OR radius[Title/abstract] OR radial[Title/abstract] OR “rotator cuff”[Title/abstract] OR sacral[Title/abstract] OR sacrum[Title/abstract] OR shin[Title/abstract] OR shins[Title/abstract] OR shoulder[Title/abstract] OR shoulders[Title/abstract] OR “soft tissue”[Title/abstract] OR spine[Title/abstract] OR spinal[Title/abstract] OR tendon[Title/abstract] OR tendons[Title/abstract] OR thigh[Title/abstract] OR thighs[Title/abstract] OR thoracic[Title/abstract] OR thorax[Title/abstract] OR thumb[Title/abstract] OR thumbs[Title/abstract] OR tibia[Title/abstract] OR tibial[Title/abstract] OR toe[Title/abstract] OR toes[Title/abstract] OR ulna[Title/abstract] OR ulnar[Title/abstract] OR “upper extremity”[Title/abstract] OR “upper extremities”[Title/abstract] OR “upper limb”[Title/abstract] OR “upper limbs”[Title/abstract] OR wrist[Title/abstract] OR wrists[Title/abstract]) AND (ache[Title/abstract] OR contusion[Title/abstract] OR contusions[Title/abstract] OR dislocation[Title/abstract] OR dislocations[Title/abstract] OR dislocated[Title/abstract] OR fracture[Title/abstract] OR fractures[Title/abstract] OR fractured[Title/abstract] OR injury[Title/abstract] OR injuries[Title/abstract] OR injured[Title/abstract] OR oedema[Title/abstract] OR edema[Title/abstract] OR orthopaedic[Title/abstract] OR orthopaedics[Title/abstract] OR orthopedic[Title/abstract] OR orthopedics[Title/abstract] OR pain[Title/abstract] OR sprain[Title/abstract] OR sprains[Title/abstract] OR sprained[Title/abstract] OR surgery[Title/abstract] OR trauma[Title/abstract] OR multitrauma[Title/abstract] OR multi-trauma[Title/abstract] OR “multi trauma”[Title/abstract])) OR “myofascial pain”[Title/abstract] OR arthralgia[Title/abstract] OR arthropathy[Title/abstract] OR arthritis[Title/abstract] OR arthritic[Title/abstract] OR backache[Title/abstract] OR myalgia[Title/abstract] OR whiplash[Title/abstract] OR “wounds and injuries”[MeSH] OR “musculoskeletal pain”[MeSH] OR arthralgia[MeSH] OR “back pain”[MeSH] OR “neck pain”[MeSH] OR “Myofascial pain syndromes”[MeSH] |
| --- | --- |

| Study design terms | randomised[Title/abstract] OR randomized[Title/abstract] OR trial[Title/abstract] OR intervention[Title/abstract] OR interventions[Title/abstract] OR randomisation[Title/abstract] OR randomization[Title/abstract] |
| --- | --- |

**Paper:** Effectiveness of interventions on work outcomes after road traffic crash-related musculoskeletal injuries: a systematic review and meta-analysis

submitted to Journal of Occupational Rehabilitation

**Authors**: Charlotte L. Brakenridge, Esther J. Smits, Elise M. Gane, Nicole E. Andrews, Gina Williams, Venerina Johnston

**Contact:** Charlotte L. Brakenridge, [c.brakenridge@uq.edu.au](mailto:c.brakenridge@uq.edu.au), The University of Queensland, School of Human Movements and Nutrition Sciences, Brisbane, QLD, Australia
