# Supplementary material for: Effectiveness of Interventions on Work Outcomes After Road Traffic Crash-Related Musculoskeletal Injuries: A Systematic Review and Meta-analysis
Source: J Occup Rehabil. 2024 Apr 5;35(1):30–47. doi: 10.1007/s10926-024-10185-z (PMC11839784; doi:10.1007/s10926-024-10185-z)
Supplement: Supplementary file 3 — Supplementary material 3 (DOCX 62 kb) [file 10926_2024_10185_MOESM3_ESM.docx]

Supplementary File 3. Intervention and comparison characteristics, work outcomes, intervention effects and analyses

| First author, year, study design [reference number] | Intervention/s | Interventionist, setting, frequency, duration | Comparison | Work Outcome  Follow up period  Intervention effect  (Bold outcomes were significantly different between groups [p<0.05]; primary and co-primary outcomes indicated) | Data analysis method for work outcome |
| --- | --- | --- | --- | --- | --- |
| Ludvigsson, 2017, 3-arm RT [59]  Lo, 2018  [35] | Therapeutic interventions  A) supervised neck-specific exercise (NSE)  B) NSE + graded exercise approach, pain management, problem solving | Physiotherapist, clinic, 2xweek, 12 weeks | C) 1 motivational interview with physiotherapist, physical examination, physical activity prescription (unsupervised) + follow up visit | Sick leave days at 12 months  A) 13.8 (43.7) vs. B) 29.7 (74.9) vs. C) 21.6 (46.5), ns  **Work Ability Index**  (scores 7-49, higher scores better)  3 months  A vs. C: -0.15, (-2.40, 2.10), p=0.90  B vs. C: +2.93, (0.35, 5.50), p=0.03  6 months  A vs. C: +2.55, (0.01, 5.09), p=0.045  B vs. C: +3.44, (0.86, 6.01), p=0.01  12 months  A vs. C: +5.74, (-3.66, 15.13), p=0.23  B vs. C: +12.31, (2.82, 21.80), p=0.01 (B also sig different from A) | One-way ANOVA, Tukey correction for post-hoc tests, intention to treat analysis  Generalised estimating equations, adjusted for age, sex, time, group × time interaction, neck load at work, PDI, HADS depression and financial situation; intention to treat analysis |
| Villafane, 2017, 2-arm non-RCT (participant choice)  [39] | Therapeutic intervention  A) cognitive behavioural exercise approach + neck collar + neck & shoulder exercises + whiplash booklet | Physiotherapist, setting NR, 15 days, participants instructed to do exercises 1/day | B) whiplash booklet + usual care (soft collar, pain killers, sick leave for 7-14 days) | % on sick leave  2 weeks  A) 0/25 (0%) vs. B) 2/16 (13%), ns  12 weeks  A) 0/25 (0%) vs. B) 0/16 (0%), ns | Chi-square, intention to treat analysis |
| Wu, 2017, 2-arm RCT  [46] | Therapeutic intervention  A) 100% increase in therapist sessions/day over usual care + weekly case conferences with in-reach rehab team | Physiotherapist and/or occupational therapist, in reach team at hospital, 2xday, duration based on clinical need, median length 6 days, median 16 sessions | B) usual care | sub-sample with musculoskeletal injuries and who were working prior to injury (n=84):  (median 4 or 6 months follow up based on injury)  % RTW  A) 20/37 (54%) vs. B) 34/47 (72%), ns  % same job out of those who RTW  A) 16/20 (80%) vs. B) 32/34 (94%), ns  % full duties out of those who RTW  A) 13/20 (65%) vs. B) 21/34 (62%), ns  % usual hours out of those who RTW  A) 13/20 (65%) vs. B) 24/34 (71%), ns | Fisher’s exact test performed on sub-sample data |
| Brooke, 2014, 2-arm non-RCT (sequential allocation)  [50] | Therapeutic intervention  A) consultation on activity limitations and participation restrictions. Case conferences with multidisciplinary team. Psychological intervention for at risk participants. Referral to physiotherapy as required. | Rehabilitation physician, outpatient hospital, 4 visits on day 10, weeks 6, 12, 18 | B) usual care | % RTW  18 weeks  A) 25/37 (68%) vs. B) 19/27 (70%), ns  **% return to normal work**  18 weeks  A) 18/37 (49%) vs. B) 7/27 (26%), p=0.018 | Cox regression for survival functions, adjusted for age and injury severity, intention to treat analysis, cases lost to follow up were included based on the last available information |
| Conforti, 2013, 2-arm RCT  [36] | Therapeutic intervention  A) laser therapy | Physiotherapist, setting NR, 5 days | B) conventional simple segmental physical rehabilitation every 3 days for 10 treatments | **Days to RTW**  End of treatment  A) 54.2 (26.7) days vs. B) 82.0 (63.2) days, p=0.0048 | T-test |
| Elbers, 2013, 2-arm RCT  [58] | Web-based intervention  A) website with information about compensation process (49 pages), 5-lesson problem-solving therapy, 10 frequently asked questions | Feedback from principle investigator, setting NR, minimal length (mean 9 minutes on website, most within 2 weeks of receiving code) | B) control website, with links to existing information | In sub-sample with whiplash  3-item Work Ability Index (1-10 VAS scale)  3 months (n=44)  A vs. B: -1.07 (-2.55, 0.41), ns  6 months (n=44)  A vs. B: -0.64 (-2.06, 0.78), ns  12 months (n=42)  A vs. B: -0.27 (-1.77, 1.23), ns | Unpaired t-test performed on sub-sample data |
| Lamb, 2013, 2-arm cluster RCT, Part 1  [32, 33] | Emergency department intervention  A) active management consultation (reassurance, encouraging return to normal activities and neck exercises, avoidance of neck collar) + ‘The Whiplash Book’ | Emergency Department (ED) clinicians, hospital, one off consultation | B) usual care | Workdays lost  4 months  A vs. B: 0 (-1.0, 1.1), p=0.880  8 months  A vs. B: 0 (-1.3, 1.6), p=0.847  12 months  A vs. B: 0 (-2.1, 1.5), p=0.888 | Multiple linear regression with bootstrapping, with adjustment for baseline employment status, WAD grade, time from attendance to ED, and clustering by ED. Missing data imputed by Multivariate Imputation by Chained Equations. Intention to treat analysis. |
| Lamb, 2013, 2-arm RCT, Part 2  [32, 33] | Therapeutic intervention  A) manual therapy, soft-tissue techniques, exercise, pain management, promoting normal activities, psychological strategies | Physiotherapist, outpatient hospital, up to 6 sessions, 8 weeks | B) reinforcement of advice given in part 1 (book or usual care pamphlet), 1 x 30-40 min session with physiotherapist | **Workdays lost**  4 months  A vs. B: -4 (-7.9, -1.1), p=0.016  8 months  A vs. B: -4 (-7.7, -0.5), p=0.018  12 months  A vs. B: -4 (-7.5, -0.02), p=0.026 | Multiple linear regression with bootstrapping, with adjustment for baseline employment status, advice at ED, baseline NDI, time from attendance to ED, and clustering by therapist. Missing data imputed by Multivariate Imputation by Chained Equations. Intention to treat analysis. |
| Schaafsma, 2012, 2-arm non-RCT  [53] | Therapeutic intervention  A) enhanced insurance consultation: up to 50% more time spent per claim, consistent communication protocol, risk screening, and prompt approval of treatment | Trained consultant, insurance company, length NR | B) usual insurance protocol | Employment status  7 months  A) 87/123 (71%) vs. B) 39/63 (62%), ns  % returned to full duties (out of those employed at 7-month follow up)  A) 73/87 (84%) vs. B) 27/39 (69%), ns | F-test or Chi-square |
| Pato, 2010, 3-arm RCT  [43] | Therapeutic compared to drug-based interventions  A) massage, learned relaxation, isometric and isotonic training of neck muscles  B) bupivacaine injection into tender point  After randomisation to A, B or C, participants were further randomised to receive CBT intervention or not:  CBT: guided by therapy manual, focus on behavioural aspects of pain, stress reduction and relaxation | A)Physiotherapist, clinic, 2xweek, 8 weeks  B)Physician, clinic, 2xweek, 8 weeks  CBT:Psychologist, clinic, 2xweek, 8 weeks | C) 1 x 200 mg flurbiprofen/day + visits with physician 2xweek for 8 weeks | Working capacity (out of 100%, mean (SD)) – Co-primary outcome  8 weeks  A) 71% (35) vs. B) 71% (40) vs. C) 68% (37), ns  CBT: 72% (32) vs. no CBT: 67% (43), ns  6 months  A) 80% (35) vs. B) 76% (35) vs. C) 76% (35), ns  CBT: 77% (32) vs. no CBT: 76% (39), ns | ANOVA |
| Amirfeyz, 2009, 2-arm non-RCT  [49] | Therapeutic intervention  A) neck posture advice and practice under direct supervision, graded activities, balance and coordination, stretching, movement and strengthening exercises | Physiotherapist, clinic, average 1xweek, 6 weeks | B) same intervention as A, but received later than 3 months post injury | Prevalence of any sick leave in past 4 weeks  6 weeks follow up  A) 19/50 (38%) vs. B) 20/76 (26%), ns | Chi-square |
| Ask, 2009, 2-arm RCT  [48] | Therapeutic intervention  A) motor control training, low loaded training program + encouraged to exercise at home | Physiotherapist, outpatient spine clinic, 1-2xweek, 6 weeks | B) endurance & strength of the neck muscles, neck & shoulder exercises, resistance exercises + encouraged to exercise at home | Prevalence of any sick leave  12 months  A) 1/10 (10%) vs. B) 6/11 (55%) | No statistical analysis reported for work outcome |
| Kongsted, 2007, 3-arm RCT  [42] | Therapeutic interventions  A) active mobilisation program  B) neck collar for 2 weeks + physiotherapy consult + active mobilisation | Physiotherapist, university research centre, 2xweek, 6 weeks for A, 4 weeks for B | C) ‘act as usual’ advice, 1-hour session. Delivered by nurse. | % of participants with sick days or reduced working hours in past month – Co-primary outcome  12 months  A) 31/140 (22%) vs. B) 40/143 (28%) vs. C) 31/123 (25%), p=0.6  RTW  12 months  A) 131/149 (88%) vs. C) 135/153 (88%) (group B not reported for full group) | Logistic regression, adjusted for age, gender, centre, baseline cervical range of motion, baseline impact of event, baseline neck pain. Intention to treat analysis. RTW outcome only raw data reported, no statistical analysis reported. |
| Ottosson, 2007, 2-arm RCT  [45] | Therapeutic intervention  A) group sessions on tissue healing, pain management, self care, relaxation exercises and posture control + educational materials | 1 session each delivered by physiotherapist, anaesthesiologist, psychologist + introductory session, outpatient hospital, 1xweek, 4 weeks | B) usual care | Weeks of sick leave  12 months  A vs. B: -3.4 weeks (-9.8, 2.9), p=0.18 | Two-sample t-test |
| Vikne, 2007, 4-arm RCT  [44] | Therapeutic intervention  A) traditional physiotherapy, home training program and 24 sling exercises for 4 months + calls every 4 months for 1 year  B) same as ‘A’ but home program continued for 1 year, adjusted once/month at institute | Physiotherapist, institute, 24 sling exercise sessions over 4 months, 3 calls over 1 year | C) same as ‘A’ with no sling exercises  D) same as ‘B’ with no sling exercises | Prevalence of any sick leave  12 months  A) 40% vs. B) 40% vs. C) 53% vs. D) 21% | Wilcoxon signed-rank tests to test within group significance. Between group significance not analysed or reported. |
| Stewart, 2007, 2-arm RCT  [38] | Therapeutic intervention  A) graded exercise sessions, CBT components + individualised program to be completed at home + advice sessions | Physiotherapist trained in CBT, physiotherapy clinic, 12 sessions over 6 weeks | B) advice sessions to resume light activity, 1 f2f consultation + 2 telephone contacts | % working at follow up  6 weeks  A) 39/66 (59%) vs. B) 52/66 (79%), ns  12 months  A) 40/63 (63%) vs. B) 46/62 (74%), ns  % returned to full duties (out of those working at follow up)  6 weeks  A) 34/39 (87%) vs. B) 41/52 (79%), ns  12 months  A) 33/40 (83%) vs. B) 39/46 (85%), ns | Logistic regression, participants omitted from analysis if follow up data missing |
| Bunketorp, 2006, 2-arm RCT  [41] | Therapeutic intervention  A) supervised training targeting neck & shoulder exercises, fear of pain & movement, increased self-efficacy for physical activities, individualised, groups of 3-4 later formed + pamphlet for reducing fear and anxiety + advice to participate in low-intensity aerobic exercise | Physiotherapist, rehabilitation centre, 2xweek for a mean of 18 sessions (range 12-42) | B) self-administered home training, 2x day; initial instructions by physiotherapist, could check with physiotherapist at rehab centre fortnightly if needed + same pamphlet & advice for aerobic exercise as ‘A’ | % with improved sick leave  3 months  A) 50% vs. B) 44%, p=0.66  9 months  A) 48% vs. B) 50%, p=0.87 | Chi-square, missing data at 9 months imputed using 3-month values |
| Scholten-Peeters, 2006, 2-arm RCT  [47] | Therapeutic intervention  A) education, advice, graded activity, exercise therapy (education and advice similar to intervention B) | Physiotherapist, clinic, 30 min sessions, max duration 9 months, mean 13 sessions over 20 weeks | B) GP care, education and advice, including graded activity, 10 min sessions. Max duration 9 months mean 4 sessions over 19 weeks. | Work activities in daily living − Co-primary outcome (VAS)  12 months  A vs. B: +11.3 (-1.0, 23.7), ns | Multiple linear regression, adjusted for neck pain intensity, work activities, gender, pre-existing problems, and high number of complaints. Analysis by intention to treat principle. Missing data imputed using group mean. |
| Sullivan, 2006, 2-arm cohort study (non-randomised)  [34] | Therapeutic intervention  A) Progressive Goal Attainment Program (PGAP), psychosocial intervention, to increase goal-directed activity & minimize psychosocial barriers. Activity monitoring & prescription, graded activity participation, cognitive restructuring. Goal is to return to work + functional restoration physical therapy. | PGAP trained physical therapists and occupational therapists, rehabilitation clinic, 1xweek, 10 weeks or until RTW | B) functional restoration physical therapy only, using a ‘sports medicine approach’. Included a RTW component but no further details given. 3 x weekly visits of 2.5 hours. Maximum of 10 weeks or until return to work | **% RTW – Primary outcome**  14 weeks  A) 75% vs. B) 50%, p<0.01 | Chi-square, last observation carried forward |
| Ferrari, 2005, 2-arm RCT  [56] | Emergency department intervention  A) one off 1-page whiplash pamphlet summarized from the Whiplash Book, delivered hospital at discharge | Research nurse, hospital, one off pamphlet | B) usual care, including generic information sheet | Employed at 3-month follow up  A) 42/49 (86%) vs. B) 42/53 (79%)  % off work at 3-month follow up  A) 5/42 (12%) vs. B) 4/42 (10%)  % with any missing workdays at 3-month follow up  A) 35/42 (83%) vs. B) 30/42 (71%) | No analysis reported for work outcomes. |
| Crawford, 2004, 2-arm RCT  [52] | Therapeutic intervention  A) advice sheet with mobilisation exercise regime, told to stop using soft collar | NR, research clinic, one off advice sheet + review at 3, 12 and 52 weeks by clinician | B) soft collar for 3 weeks + same exercise regime advice sheet as ‘A’ | **Days to RTW**  12 months  A: 17.3 (95%CI: 11.7-23) vs. B: 34.4 (95%CI: 13.9-55), p=0.03 | Unpaired t-test |
| Ventegodt, 2004, 2-arm RCT  [54] | Therapeutic intervention  A) 2 days of teachings in philosophy of life + 6-10 individual sessions in gestalt psychotherapy and body therapy (Rosen therapy and Cranio Sacral therapy) + 1 day course 2 months later to close the intervention | NR, research centre, 3 days + 6-10 sessions, 2 months | B) non-treated control | Sick leave  3 months  A) 8/26 (31%) vs. B) 4/29 (14%), p=0.19 | Chi-square |
| Rosenfeld, 2003, 4-arm RCT (2-arm combined in analysis)  [37] | Therapeutic intervention  A) postural control and cervical rotation exercises prescribed, to be completed daily at home. Participants taught to identify new or increased symptoms. Intervention initiated within 96 hours of injury or after 14 days (intervention groups combined). If symptoms persisted > 20 days, re-evaluation and treatment using McKenzie principles. | Physiotherapist, setting NR, mean 4 sessions, 6 weeks or earlier if symptoms resolved | B) leaflet received within 96 hours or after 14 days with information on injury (groups combined) mechanisms, suitable activities and postural correction, advice to use soft collar and perform active movements 2-3x daily after a few weeks. | Sick leave days in preceding 6 months  6 months  A) 13.3 (39.6) vs. B) 19.4 (39.6), p>0.05  **3 years**  A) 10.6 (42.4) vs. B) 31.7 (62.8), p=0.03 | Friedmann’s test. Analysis by intention to treat principle. |
| Bonk, 2000, 2-arm RCT  [40] | Therapeutic intervention  A) mobilisation of the neck + active mobilisation by the participant + strength and isometric exercises + muscle strengthening exercises + advice to maintain normal neck posture and avoid collar | Physiotherapist, setting NR, 7 sessions, 3 weeks | B) collar use for 3 weeks | % with any work missed  A) 15/47 (32%) vs. B) 22/50 (44%) | No analysis reported for work outcome |
| Borchgrevink, 1998, 2-arm RCT  [55] | Emergency department intervention  A) act as usual advice + instructions in self-training of the neck | ED clinician, hospital, one off advice | B) soft neck collar for 14 days + 14 days of sick leave + instructions in self-training of the neck | % with any sick leave post 14 days  6 months  A) 24/82 (33%) vs. B) 21/96 (22%), ns  % on 100% sick leave at 6 months  A) 2/82 (2%) vs. B) 2/96 (2%), ns  % on 50% sick leave at 6 months  A) 6/82 (7%) vs. B) 5/96 (5%), ns | Chi-square |
| Pettersson, 1998, 2-arm RCT  [57] | Drug-based intervention  A) high dose methylprednisolone administered within 8 hours after injury | Hospital pharmacist, hospital, one off treatment | B) placebo, delivered by hospital pharmacist  (all participants received soft collar for 1-2 weeks, physiotherapy and analgesics) | **Sick days – Co-primary outcome**  6 months  A) 4.7 (9.2) vs. B) 51.7 (72.8), p=0.0097  **% on sick leave due to WAD – Co-primary outcome**  A) 0/20 (0%) vs. B) 4/19 (21%), p=0.047 (p value 0.053 if 1 missing person counted as not on sick leave)  **Sick leave profile**  A vs. B, p=0.003 | Wilcoxon’s rank sum test for independent samples for continuous data.  Fisher’s exact test for categorical data. Survival analysis for sick leave profile. Means and SDs calculated using raw data (from figure 2). |
| Provinciali, 1996, 2-arm RCT  [51] | Therapeutic intervention  A) relaxation training, postural training, manual treatment of the spine, psychological support, eye fixation exercises to prevent dizziness + soft collar | NR, clinic, 10 sessions, 2 weeks | B) intervention focused on physical agents including TENS machine, pulsed electromagnetic therapy, ultrasound + soft collar | **Days to return to work**  6 months  A) 38.4 (10.5) vs. 54.3 (18.4), p<0.001  In usual occupation at 6 months  A) 29/30 (97%) vs. B) 24/30 (80%) | One-way ANOVA for continuous outcome, no analysis reported for categorical outcome |

When only percentage of participants was reported and not number of participants, author CLB calculated the number of participants (i.e., % x sample size).

CBT = cognitive behaviour therapy, DASS = The Depression, Anxiety and Stress Scale, GP = general practitioner, HADS = The Hospital Anxiety and Depression Scale, NDI = Neck Disability Index, NR = not reported, ns = not significant, OMPQ = Örebro Musculoskeletal Pain Questionnaire, PSFS = Patient Specific Functional Scale , RCT = randomised controlled trial, SCL-90 = The Symptom Checklist-90, SD = standard deviation, SF = Short Form, TENS = transcutaneous electrical nerve stimulation, TSQ = Trauma Screening Questionnaire, VAS = visual analogue scale, WAD = whiplash associated disorder

**Paper:** Effectiveness of interventions on work outcomes after road traffic crash-related musculoskeletal injuries: a systematic review and meta-analysis, submitted to Journal of Occupational Rehabilitation

**Authors**: Charlotte L. Brakenridge, Esther J. Smits, Elise M. Gane, Nicole E. Andrews, Gina Williams, Venerina Johnston

**Contact:** Charlotte L. Brakenridge, [c.brakenridge@uq.edu.au](mailto:c.brakenridge@uq.edu.au), The University of Queensland, School of Human Movements and Nutrition Sciences, Brisbane, QLD, Australia
