# Supplementary material for: Effectiveness of Interventions on Work Outcomes After Road Traffic Crash-Related Musculoskeletal Injuries: A Systematic Review and Meta-analysis
Source: J Occup Rehabil. 2024 Apr 5;35(1):30–47. doi: 10.1007/s10926-024-10185-z (PMC11839784; doi:10.1007/s10926-024-10185-z)
Supplement: Supplementary file 4 — Supplementary material 4 (DOCX 18.0 kb) [file 10926_2024_10185_MOESM4_ESM.docx]

Supplementary File 4. Excluded papers at the full text stage

| **First author and year, or trial number** | **Reason for exclusion** |
| --- | --- |
| Andersson, A. L., (2005). | Not adults with musculoskeletal injury from road traffic crash. Sample described using ISS, not injury type, unable to get injury type data |
| Beer, A., (2012). | Not adults with musculoskeletal injury from road traffic crash. Not from RTC specifically. |
| Christensen, M. C., (2011). | Not adults with musculoskeletal injury from road traffic crash. Blunt/penetrating trauma not musculoskeletal specifically. |
| Classen, S., (2017). | Not adults with musculoskeletal injury from road traffic crash. Combat veterans with polytrauma. |
| Ehrenborg, C. (2010). | Not adults with musculoskeletal injury from road traffic crash. Not from RTC specifically and no information about cause of injury. |
| Faux, S. G., (2015). | Not adults with musculoskeletal injury from road traffic crash. Not musculoskeletal specifically. |
| Franzen, C., (2009). | Not adults with musculoskeletal injury from road traffic crash |
| Ganesh, G. S., (2015). | Not adults with musculoskeletal injury from road traffic crash |
| Huang, Y. Y., (2021). | Not adults with musculoskeletal injury from road traffic crash |
| Janssen, C., (2009). | Not adults with musculoskeletal injury from road traffic crash |
| Khan, I., (2020). | Not adults with musculoskeletal injury from road traffic crash |
| Klobas, L., (2006). | Not adults with musculoskeletal injury from road traffic crash |
| Larsman, P., (2010). | Not adults with musculoskeletal injury from road traffic crash |
| Miller, J., (2015). | Not adults with musculoskeletal injury from road traffic crash |
| Moll, L. T., (2018). | Not adults with musculoskeletal injury from road traffic crash |
| Ortego, G., (2022). | Not adults with musculoskeletal injury from road traffic crash |
| Peolsson, A., (2022). | Not adults with musculoskeletal injury from road traffic crash |
| Peterson, G., (2018). | Not adults with musculoskeletal injury from road traffic crash |
| Porritt, D., (1980). | Not adults with musculoskeletal injury from road traffic crash. Injury types not reported. |
| Schermer, C. R., (2006). | Not adults with musculoskeletal injury from road traffic crash. Patients had DUI, admitted to hospital, no mention of injury. |
| Simunovic, Z., (2000). | Not adults with musculoskeletal injury from road traffic crash |
| Sommers, M. S., (2006). | Not adults with musculoskeletal injury from road traffic crash |
| Wood, K. B., (2015). | Not adults with musculoskeletal injury from road traffic crash. Only 45% of cohort from RTCs, results not reported separately. |
| Zoega, B., (2000). | Not adults with musculoskeletal injury from road traffic crash. Only 40% RTC, results not reported separately. |
| NCT02060734 | Not adults with musculoskeletal injury from road traffic crash. Whiplash associated disorder after injury, not RTC specific, trial has been withdrawn. |
| NCT02692937 | Not adults with musculoskeletal injury from road traffic crash. Whiplash associated disorder after injury, not RTC specific. |
| NCT02764983 | Not adults with musculoskeletal injury from road traffic crash. Participants had traumatic brain injury. |
| NCT03036007 | Not adults with musculoskeletal injury from road traffic crash. Patients have chronic neck pain, but not specifically from RTC. |
| Bohman, T. (2022). | No comparison/ control group |
| Collie, A., (2015). | No comparison/ control group |
| Ellis, N., (2010). | No comparison/ control group. Study downscaled to phase 1 feasibility study due to problems with recruitment. |
| Verma, S., (2022). | No comparison/ control group |
| Cote, P., (2008). | No work outcome. Work status reported as measure in protocol paper but not in main outcomes paper. Collection of work data ceased during the study due to issues with questionnaire completion. |
| DeLomba, W., (2021). | No work outcome |
| Elphinston, R. A., (2020). | No work outcome |
| Fitz-Ritson, D. (1994). | No work outcome |
| Kongsted, A., (2008). | No work outcome. Recovery outcome included both work and pain. |
| Nystrom, B., (2016). | No work outcome |
| Seferiadis, A., (2016). | No work outcome |
| Suissa, S., (2006). | No work outcome |
| Wicksell, R. K., (2008). | No work outcome |
| Peolsson, A., (2017). | Study protocol/clinical trial protocol only |
| Peterson, G., (2022). | Study protocol/clinical trial protocol only |
| Rebbeck, T., (2016). | Study protocol/clinical trial protocol only |
| NCT05593289 | Study protocol/clinical trial protocol only |
| NCT05704023 | Study protocol/clinical trial protocol only |
| NCT01699334 | Study protocol/clinical trial protocol only |
| NCT03022812 | Study protocol/clinical trial protocol only |
| ACTRN12621000030875 | Study protocol/clinical trial protocol only |

**Paper:** Effectiveness of interventions on work outcomes after road traffic crash-related musculoskeletal injuries: a systematic review and meta-analysis, submitted to Journal of Occupational Rehabilitation

**Authors**: Charlotte L. Brakenridge, Esther J. Smits, Elise M. Gane, Nicole E. Andrews, Gina Williams, Venerina Johnston

**Contact:** Charlotte L. Brakenridge, [c.brakenridge@uq.edu.au](mailto:c.brakenridge@uq.edu.au), The University of Queensland, School of Human Movements and Nutrition Sciences, Brisbane, QLD, Australia
