# Supplementary material for: Effectiveness of Interventions on Work Outcomes After Road Traffic Crash-Related Musculoskeletal Injuries: A Systematic Review and Meta-analysis
Source: J Occup Rehabil. 2024 Apr 5;35(1):30–47. doi: 10.1007/s10926-024-10185-z (PMC11839784; doi:10.1007/s10926-024-10185-z)
Supplement: Supplementary file 5 — Supplementary material 5 (DOCX 16.2 kb) [file 10926_2024_10185_MOESM5_ESM.docx]

Supplementary File 5. Subgroup analyses

| **Work outcome** | **Subgroup analysis** | **k** | **Pooled effect (mean difference and 95%CI or risk ratio and 95%CI)** | **Heterogeneity** |
| --- | --- | --- | --- | --- |
| % returned to work or employed | Intervention vs. usual care/control | 4 | RR = 1.01 (0.86, 1.19) | Tau^2^ = 0.01, I^2^ = 36%, Chi^2^ = 4.67, p=0.20 |
| % returned to work or employed | Intervention vs. intervention | 4 | RR = 1.07 (0.86, 1.33) | Tau^2^ = 0.04, I^2^ = 83%, Chi^2^ = 17.25, p<0.001 |
| Days of sick leave | Intervention vs. usual care/control | 3 | -20.35 days (-53.30, 12.60) | Tau^2^ = 632.91, I^2^ = 78%, Chi^2^ = 8.90, p=0.01 |
| Days of sick leave | Intervention vs. intervention | 4 | -3.98 days (-7.25, -0.72) | Tau^2^ = 0.00, I^2^ = 0%, Chi^2^ = 1.02, p=0.80 |
| % with sick leave | Intervention vs. usual care/control | 3 | RR = 1.15 (0.53, 2.51) | Tau^2^ = 0.23, I^2^ = 46%, Chi^2^ = 3.72, p=0.16 |
| % with sick leave | Intervention vs. intervention | 7 | RR = 1.00 (0.73, 1.37) | Tau^2^ = 0.07, I^2^ = 40%, Chi^2^ = 9.98, p=0.13 |
| Standardised effects | Intervention vs. usual care/control | 8 | SMD = -0.07 (-0.30, 0.16) | Tau^2^ = 0.05, I^2^ = 54%, Chi^2^ = 15.21, p=0.03 |
| Standardised effects | Intervention vs. intervention | 16 | SMD = -0.18 (-0.37, 0.01) | Tau^2^ = 0.09, I^2^ = 69%, Chi^2^ = 48.80, p<0.001 |

**Paper:** Effectiveness of interventions on work outcomes after road traffic crash-related musculoskeletal injuries: a systematic review and meta-analysis, submitted to Journal of Occupational Rehabilitation

**Authors**: Charlotte L. Brakenridge, Esther J. Smits, Elise M. Gane, Nicole E. Andrews, Gina Williams, Venerina Johnston

**Contact:** Charlotte L. Brakenridge, [c.brakenridge@uq.edu.au](mailto:c.brakenridge@uq.edu.au), The University of Queensland, School of Human Movements and Nutrition Sciences, Brisbane, QLD, Australia
