# Supplementary material for: Effectiveness of Interventions on Work Outcomes After Road Traffic Crash-Related Musculoskeletal Injuries: A Systematic Review and Meta-analysis
Source: J Occup Rehabil. 2024 Apr 5;35(1):30–47. doi: 10.1007/s10926-024-10185-z (PMC11839784; doi:10.1007/s10926-024-10185-z)
Supplement: Supplementary file 6 — Supplementary material 6 (DOCX 23.2 kb) [file 10926_2024_10185_MOESM6_ESM.docx]

Supplementary File 6

Table 1. Sensitivity analysis for days to return to work

| **Days to return to work** | **Pooled effect (mean difference and 95%CI)** | **k** |
| --- | --- | --- |
| Original result | −17.84 days (−24.94, −10.74) | 3 |
| Result minus Conforti 2013 | −16.06 days (−23.77, −8.36) | 2 |
| Result minus Crawford 2004 | −18.96 days (−29.14, −8.77) | 2 |
| Result minus Provinciali 1996 | −23.15 days (−36.88, −9.42) | 2 |

Table 2. Sensitivity analysis for percentage of participants returned to work or employed at follow up

| **% returned to work** | **Pooled effect (risk ratio and 95%CI)** | **k** |
| --- | --- | --- |
| Original result | RR = 1.03 (0.91, 1.18) | 8 |
| Result minus Wu 2017 | RR = 1.07 (0.94, 1.21) | 7 |
| Result minus Stewart 2007 | RR = 1.08 (0.96, 1.22) | 7 |
| Result minus Brooke 2014 | RR = 1.04 (0.91, 1.20) | 7 |
| Result minus Kongsted 2007 | RR = 1.04 (0.88, 1.24) | 7 |
| Result minus Ferrari 2005 | RR = 1.03 (0.88, 1.20) | 7 |
| Result minus Schaafsma 2012 | RR = 1.02 (0.88, 1.18) | 7 |
| Result minus Provinciali 1996 | RR = 1.01 (0.88, 1.16) | 7 |
| Result minus Sullivan 2006 | RR = 1.00 (0.88, 1.12) | 7 |

Table 3. Sensitivity analysis for days of sick leave

| **Days of sick leave** | **Pooled effect (mean difference and 95%CI)** | **k** |
| --- | --- | --- |
| Original result | −3.27 days (−8.11, 1.56) | 7 |
| Result minus Pettersson 1998 | −1.69 days (−4.60, 1.21) | 6 |
| Result minus Ottosson 2007 | −3.04 days (−7.90, 1.83) | 6 |
| Result minus Ludvigsson NSE 2017 | −3.14 days (−8.25, 1.98) | 6 |
| Result minus Ludvigsson NSEB 2017 | −3.77 days (−8.84, 1.30) | 6 |
| Result minus Rosenfeld 2003 | −3.16 days (−8.38, 2.06) | 6 |
| Result minus Lamb step 1 2013 | −7.60 days (−17.17, 1.98) | 6 |
| Result minus Lamb step 2 2013 | −6.69 days (−17.53, 4.15) | 6 |

Table 4. Sensitivity analysis for percentage of participants with sick leave

| **% sick leave** | **Pooled effect (risk ratio and 95%CI)** | **k** |
| --- | --- | --- |
| Original result | RR = 1.06 (0.82, 1.36) | 10 |
| Result minus Pettersson 1998 | RR = 1.08 (0.86, 1.36) | 9 |
| Result minus Villafane 2017 | RR = 1.08 (0.85, 1.37) | 9 |
| Result minus Ask 2009 | RR = 1.09 (0.88, 1.37) | 9 |
| Result minus Bonk 2000 | RR = 1.12 (0.87, 1.45) | 9 |
| Result minus Kongsted active intervention 2007 | RR = 1.09 (0.82, 1.44) | 9 |
| Result minus Kongsted neck collar 2007 | RR = 1.03 (0.76, 1.39) | 9 |
| Result minus Ferrari 2005 | RR = 1.01 (0.72, 1.40) | 9 |
| Result minus Borchgrevink 1998 | RR = 1.01 (0.75, 1.35) | 9 |
| Result minus Amirfeyz 2009 | RR = 1.00 (0.75, 1.33) | 9 |
| Result minus Ventegodt 2004 | RR = 1.02 (0.79, 1.33) | 9 |

Table 5. Sensitivity analysis for percentage of participants returning to full or normal duties out of those who had returned to work

| **% full or normal duties** | **Pooled effect (risk ratio and 95%CI)** | **k** |
| --- | --- | --- |
| Original result | RR = 1.17 (1.01, 1.36) | 4 |
| Result minus Wu 2017 | RR = 1.21 (0.98, 1.49) | 3 |
| Result minus Stewart 2007 | RR = 1.24 (0.97, 1.60) | 3 |
| Result minus Schaafsma 2012 | RR = 1.19 (0.90, 1.58) | 3 |
| Result minus Brooke 2014 | RR = 1.14 (0.99, 1.30) | 3 |

Table 6. Sensitivity analysis for standardised effects

| **Standardised effects** | **Pooled effect (standardised mean difference and 95%CI)** | **k** |
| --- | --- | --- |
| Original result | −0.14 (−0.25, −0.03) | 24 |
| Result minus Provinciali 1996 | −0.10 (−0.20, −0.01) | 23 |
| Result minus Pettersson 1998 | −0.12 (−0.22, −0.01) | 23 |
| Result minus Conforti 2013 | −0.10 (−0.21, −0.00) | 23 |
| Result minus Ask 2009 | −0.13 (−0.25, −0.02) | 23 |
| Result minus Villafane 2017 | −0.13 (−0.25, −0.02) | 23 |
| Result minus Crawford 2004 | −0.13 (−0.24, −0.01) | 23 |
| Result minus Sullivan 2006 | −0.13 (−0.24, −0.01) | 23 |
| Result minus Ottosson 2007 | −0.13 (−0.25, −0.02) | 23 |
| Result minus Ludvigsson NSE 2017 | −0.13 (−0.25, −0.02) | 23 |
| Result minus Ludvigsson NSEB 2017 | −0.15 (−0.26, −0.03) | 23 |
| Result minus Lamb step 1 2013 | −0.15 (−0.27, −0.03) | 23 |
| Result minus Lamb step 2 2013 | −0.14 (−0.26, −0.01) | 23 |
| Result minus Rosenfeld 2003 | −0.14 (−0.25, −0.02) | 23 |
| Result minus Bonk 2000 | −0.14 (−0.25, −0.02) | 23 |
| Result minus Ferrari 2005 | −0.14 (−0.25, −0.02) | 23 |
| Result minus Schaafsma 2012 | −0.14 (−0.26, −0.02) | 23 |
| Result minus Kongsted active intervention 2007 | −0.14 (−0.26, −0.03) | 23 |
| Result minus Kongsted neck collar 2007 | −0.14 (−0.26, −0.03) | 23 |
| Result minus Brooke 2014 | −0.14 (−0.25, −0.03) | 23 |
| Result minus Borchgrevink 1998 | −0.15 (−0.26, −0.03) | 23 |
| Result minus Amirfeyz 2009 | −0.15 (−0.26, −0.03) | 23 |
| Result minus Wu 2017 | −0.15 (−0.26, −0.03) | 23 |
| Result minus Ventegodt 2004 | −0.14 (−0.25, −0.03) | 23 |
| Result minus Stewart 2007 | −0.15 (−0.26, −0.04) | 23 |

**Paper:** Effectiveness of interventions on work outcomes after road traffic crash-related musculoskeletal injuries: a systematic review and meta-analysis, submitted to Journal of Occupational Rehabilitation

**Authors**: Charlotte L. Brakenridge, Esther J. Smits, Elise M. Gane, Nicole E. Andrews, Gina Williams, Venerina Johnston

**Contact:** Charlotte L. Brakenridge, [c.brakenridge@uq.edu.au](mailto:c.brakenridge@uq.edu.au), The University of Queensland, School of Human Movements and Nutrition Sciences, Brisbane, QLD, Australia
