# Supplementary material for: Effectiveness of Interventions on Work Outcomes After Road Traffic Crash-Related Musculoskeletal Injuries: A Systematic Review and Meta-analysis
Source: J Occup Rehabil. 2024 Apr 5;35(1):30–47. doi: 10.1007/s10926-024-10185-z (PMC11839784; doi:10.1007/s10926-024-10185-z)
Supplement: Supplementary file 7 — Supplementary material 7 (DOCX 22.2 kb) [file 10926_2024_10185_MOESM7_ESM.docx]

Supplementary File 7. Univariate meta-regression model of standardised sick leave and return to work outcomes with participant, intervention and external characteristics as predictors

| Characteristic | n | Standardised Mean Difference (95%CI) | p |
| --- | --- | --- | --- |
| *Participant characteristics* |  |  |  |
| Injury type |  |  |  |
| Mixed | 4 | -0.02 (-0.47, 0.51) | 0.454 |
| Whiplash only | 20 | -0.27 (-0.52, -0.02) |  |
| Whiplash injury severity |  |  |  |
| Including grade 3 whiplash | 7 | -0.04 (-0.46, 0.38) | 0.174 |
| No grade 3 whiplash | 11 | -0.41 (-0.73, -0.08) |  |
|  |  |  |  |
| *Intervention characteristics* |  |  |  |
| Therapeutic intervention |  |  |  |
| Yes | 20 | -0.22 (-0.46, 0.03) | 0.971 |
| No | 4 | -0.23 (-1.00, 0.54) |  |
| Interventionist profession |  |  |  |
| Physiotherapist | 15 | -0.22 (-0.52, 0.07) | 0.643 |
| Other | 9 | -0.21 (-0.60, 0.18) |  |
| Mixed professional team |  |  |  |
| Yes  No | 4  20 | -0.08 (-0.80, 0.64)  -0.25 (-0.49, 0.00) | 0.844 |
| Intervention setting |  |  |  |
| Hospital | 8 | -0.10 (-0.43, 0.23) | 0.525 |
| Other | 16 | -0.28 (-0.58, 0.02) |  |
| Supervised component |  |  |  |
| Yes | 15 | -0.20 (-0.54, 0.13) | 0.464 |
| No | 9 | -0.25 (-0.51, 0.02) |  |
|  |  |  |  |
| *External characteristics* |  |  |  |
| Location |  |  |  |
| Australia | 4 | 0.20 (-0.34, 0.75) | 0.074 |
| Scandinavia | 10 | -0.19 (-0.58, 0.20) | 0.647 |
| Other Europe | 8 | -0.42 (-0.84, 0.01) | 0.216 |
| Canada | 2 | -0.44 (-2.84, 1.96) | 0.276 |
|  |  |  |  |
| *Measurement characteristics* |  |  |  |
| Primary or co-primary work outcome |  |  |  |
| Yes | 5 | -0.52 (-1.13, 0.09) | 0.057 |
| No | 19 | -0.14 (-0.38, 0.10) |  |
| Measurement of outcome  Continuous  Categorical | 10  14 | -0.35 (-0.62, -0.07)  -0.13 (-0.47, 0.21) | 0.080 |
| Type of outcome |  |  |  |
| Return to work / employment | 9 | -0.23 (-0.62, 0.17) | 0.414 |
| Sick leave | 15 | -0.21 (-0.51, 0.08) |  |
|  |  |  |  |
|  | n | Coefficient (95%CI) | p |
| *Participant characteristics* |  |  |  |
| % women | 24 | 0.006 (-0.010, 0.023) | 0.418 |
| Mean or median age | 23 | 0.018 (-0.025, 0.060) | 0.396 |
| Time since injury or hospital admission^a^ | 21 | 0.001 (-0.000, 0.001) | 0.094 |
|  |  |  |  |
| *Intervention characteristics* |  |  |  |
| Duration of intervention (days) | 23 | 0.003 (-0.003, 0.008) | 0.380 |
| Duration of follow up (days)^b^ | 23 | -0.000 (-0.002, 0.001) | 0.666 |
| Number of intervention contacts^c^ | 22 | 0.013 (-0.016, 0.041) | 0.378 |

^a^ mean or median if reported, otherwise eligibility criteria (e.g., within 2 days of injury) or duration of symptoms

^b^ planned follow up length or mean if reported

^c^ mean if reported, otherwise number of planned contacts

Additional predictors were considered but were not evaluated due to lack of variance across studies (e.g. compensation scheme (23/24 fault based)), or insufficient studies with data on predictor (e.g. workplace characteristics (none reported), or proportion of participants compensated (only reported by 2 studies)).

**Paper:** Effectiveness of interventions on work outcomes after road traffic crash-related musculoskeletal injuries: a systematic review and meta-analysis, submitted to Journal of Occupational Rehabilitation

**Authors**: Charlotte L. Brakenridge, Esther J. Smits, Elise M. Gane, Nicole E. Andrews, Gina Williams, Venerina Johnston

**Contact:** Charlotte L. Brakenridge, [c.brakenridge@uq.edu.au](mailto:c.brakenridge@uq.edu.au), The University of Queensland, School of Human Movements and Nutrition Sciences, Brisbane, QLD, Australia
