# Supplementary material for: Effectiveness of Interventions on Work Outcomes After Road Traffic Crash-Related Musculoskeletal Injuries: A Systematic Review and Meta-analysis
Source: J Occup Rehabil. 2024 Apr 5;35(1):30–47. doi: 10.1007/s10926-024-10185-z (PMC11839784; doi:10.1007/s10926-024-10185-z)
Supplement: Supplementary file 8 — Supplementary material 8 (DOCX 18.8 kb) [file 10926_2024_10185_MOESM8_ESM.docx]

Supplementary File 8.

Table 1. Meta-analyses for health and functional-related outcomes across studies, and in those with a significant work outcome

| **Health and functional-related outcome** | **Pooled effect (mean difference and 95%CI or risk ratio and 95%CI)** | **k** | **Heterogeneity** | **Publication bias** | **Pooled effect in studies with significant work outcome** | **k** |
| --- | --- | --- | --- | --- | --- | --- |
| Pain intensity (VAS) (out of 100) | -6.17 units (-11.96, -0.39) | 14 | Tau^2^ = 94.19, I^2^ = 86%, Chi^2^ = 94.88, p<0.001 | None detected | -3.43 units (-13.50, 6.64) | 5 |
| Neck disability index (out of 50) | -1.77 units (-3.24, -0.30) | 7 | Tau^2^ = 2.72, I^2^ = 76%, Chi^2^ = 24.76, p<0.001 | None detected | -2.01 units (-3.02, -0.99) | 2 |
| Prevalence of neck pain | RR = 0.50 (0.11, 2.32) | 3 | Tau^2^ = 1.64, I^2^ = 92%, Chi^2^ = 23.70, p<0.001 | None detected | Insufficient data, only 1 study | 1 |
| SF Physical health-related quality of life (out of 100) | 1.40 units (-0.05, 2.85) | 6 | Tau^2^ = 1.70, I^2^ = 56%, Chi^2^ = 11.24, p=0.05 | None detected | 0.50 units (-0.95, 1.95) | 2 |
| SF Mental health-related quality of life (out of 100) | 1.41 units (-0.17, 2.99) | 6 | Tau^2^ = 1.52, I^2^ = 43%, Chi^2^ = 8.77, p=0.12 | None detected | 1.40 units (-0.57, 3.38) | 2 |
| Self-reported recovery | RR = 1.06 (0.96, 1.17) | 7 | Tau^2^ = 0.01, I^2^ = 64%, Chi^2^ = 16.78, p=0.01 | None detected | Insufficient data, only 1 study | 1 |

VAS = visual analogue scale, SF = Short-Form

Table 2. List of remaining health and functional-related outcomes and number with a significant intervention effect

| **Health and functional-related outcome** | **List of outcomes and number with significant effect** |
| --- | --- |
| Physical functioning | PSFS (2/2 studies) ^a^  Cervical or neck range of motion (0/7 studies) ^b^  FIM (0/1 study)  SMFA (0/1 study)  Timed up and go test (0/1 study)  % of participants functionally recovered (1/1 study) |
| Psychological functioning | Pain catastrophising (1/1 study) ^a^  Self-reported wellbeing (1/1 study)  Global perceived effect (1/1 study)  Kinesiophobia (1/3 studies) ^c^  Depression or anxiety (0/6 studies) ^d^  PTSD (0/1 study) ^a^  Coping capability (0/1 study) |
| Cognitive functioning | Concentration problems (0/1 study) ^a^  CFQ (0/1 study) |
| Return to activities | Return to usual activities (1/1 study)  Return to driving (0/1 study) ^a^ |
| Pain-related disability (not NDI) | PDI (0/1 study) ^a^  CNFDS (0/1 study)  Bournemouth Questionnaire (0/1 study)  OMPQ (0/1 study)  Roland and Morris disability score (0/1 study) |

PSFS = Patient Specific Functional Scale, FIM = Functional Independence Measure, SMFA = Short Musculoskeletal Function Assessment, PTSD = post traumatic stress disorder, CFQ = Cognitive Failures Questionnaire, NDI = Neck Disability Index, PDI = Pain Disability Index, CNFDS = Copenhagen Neck Functional Disability Scale, OMPQ = Örebro Musculoskeletal Pain Questionnaire

^a^ 1 study had significant work outcome

^b^ 2 studies had significant work outcomes

^c^ 1 study with non-significant effects for Kinesiophobia had a significant effect for a work outcome

^d^ 3 studies had significant work outcomes

**Paper:** Effectiveness of interventions on work outcomes after road traffic crash-related musculoskeletal injuries: a systematic review and meta-analysis, submitted to Journal of Occupational Rehabilitation

**Authors**: Charlotte L. Brakenridge, Esther J. Smits, Elise M. Gane, Nicole E. Andrews, Gina Williams, Venerina Johnston

**Contact:** Charlotte L. Brakenridge, [c.brakenridge@uq.edu.au](mailto:c.brakenridge@uq.edu.au), The University of Queensland, School of Human Movements and Nutrition Sciences, Brisbane, QLD, Australia
