# Supplementary material for: Effectiveness of Interventions on Work Outcomes After Road Traffic Crash-Related Musculoskeletal Injuries: A Systematic Review and Meta-analysis
Source: J Occup Rehabil. 2024 Apr 5;35(1):30–47. doi: 10.1007/s10926-024-10185-z (PMC11839784; doi:10.1007/s10926-024-10185-z)
Supplement: Supplementary file 9 — Supplementary material 9 (DOCX 146.8 kb) [file 10926_2024_10185_MOESM9_ESM.docx]

Supplementary File 9.

Table 1. Risk of Bias Table for Randomised Trials

|  | Randomisation process | Deviations from intended interventions | Missing outcome data | Measurement of the outcome | Selection of the reported result | Overall |
| --- | --- | --- | --- | --- | --- | --- |
| Ludvigsson 2017 |  |  |  |  |  |  |
| Wu 2017 |  |  |  |  |  |  |
| Conforti 2013 |  |  |  |  |  |  |
| Elbers 2013 |  |  |  |  |  |  |
| Lamb 2013 part 1* | / |  |  |  |  |  |
| Lamb 2013 part 2 |  |  |  |  |  |  |
| Pato 2010 |  |  |  |  |  |  |
| Ask 2009 |  |  |  |  |  |  |
| Kongsted 2007 |  |  |  |  |  |  |
| Ottosson 2007 |  |  |  |  |  |  |
| Vikne 2007 |  |  |  |  |  |  |
| Stewart 2007 |  |  |  |  |  |  |
| Bunketorp 2006 |  |  |  |  |  |  |
| Scholten-Peeters 2006 |  |  |  |  |  |  |
| Ferrari 2005 |  |  |  |  |  |  |
| Crawford 2004 |  |  |  |  |  |  |
| Ventegodt 2004 |  |  |  |  |  |  |
| Rosenfeld 2003 |  |  |  |  |  |  |
| Bonk 2000 |  |  |  |  |  |  |
| Borchgrevink 1998 |  |  |  |  |  |  |
| Pettersson 1998 |  |  |  |  |  |  |
| Provinciali 1996 |  |  |  |  |  |  |

|  | Low risk |
| --- | --- |
|  | Some concerns |
|  | High risk |

* Lamb 2013 Step 1 used a cluster randomised design so the ‘Revised Cochrane risk of bias tool for randomized trials (RoB 2.0): Additional considerations for cluster randomized trials’ checklist was used for Part 1 of the trial (the cluster randomised component). For this checklist the Randomisation process is divided into Part A ‘Risk of bias arising from the randomisation process in a cluster-randomised trial’ and Part B ‘Risk of bias arising from the timing of identification and recruitment of participants in a cluster-randomised trial’, as such both bias ratings are presented in this column.

Table 2. Risk of Bias Table for Non-randomised Trials

|  | Confounding | Participant selection | Intervention classification | Deviations from intended interventions | Missing data | Measurement of outcomes | Selection of the reported result | Overall |
| --- | --- | --- | --- | --- | --- | --- | --- | --- |
| Villafane 2017 |  |  |  |  |  |  |  |  |
| Brooke 2014 |  |  |  |  |  |  |  |  |
| Schaafsma 2012 |  |  |  |  |  |  |  |  |
| Amirfeyz 2009 |  |  |  |  |  |  |  |  |
| Sullivan 2006 |  |  |  |  |  |  |  |  |

|  | Low risk |
| --- | --- |
|  | Moderate risk |
|  | Serious risk |
|  | Critical risk |
|  | No information |

**Paper:** Effectiveness of interventions on work outcomes after road traffic crash-related musculoskeletal injuries: a systematic review and meta-analysis, submitted to Journal of Occupational Rehabilitation

**Authors**: Charlotte L. Brakenridge, Esther J. Smits, Elise M. Gane, Nicole E. Andrews, Gina Williams, Venerina Johnston

**Contact:** Charlotte L. Brakenridge, [c.brakenridge@uq.edu.au](mailto:c.brakenridge@uq.edu.au), The University of Queensland, School of Human Movements and Nutrition Sciences, Brisbane, QLD, Australia
