# Supplementary material for: Effectiveness of Interventions on Work Outcomes After Road Traffic Crash-Related Musculoskeletal Injuries: A Systematic Review and Meta-analysis
Source: J Occup Rehabil. 2024 Apr 5;35(1):30–47. doi: 10.1007/s10926-024-10185-z (PMC11839784; doi:10.1007/s10926-024-10185-z)
Supplement: Supplementary file 10 — Supplementary material 10 (DOCX 19.7 kb) [file 10926_2024_10185_MOESM10_ESM.docx]

Supplementary File 10. GRADE for outcomes

Question: Intervention compared to Comparison for musculoskeletal injury after road traffic crash

| **Certainty assessment** | | | | | | | **№ of patients** | | **Effect** | | **Certainty** | **Importance** |
| --- | --- | --- | --- | --- | --- | --- | --- | --- | --- | --- | --- | --- |
| **№ of studies** | **Study design** | **Risk of bias** | **Inconsistency** | **Indirectness** | **Imprecision** | **Other considerations** | **Intervention** | **Comparison** | **Relative (95% CI)** | **Absolute (95% CI)** |  |  |
| **Days to return to work (follow-up: range 6 months to 12 months)** | | | | | | | | | | | | |
| 3 | randomised trials | very serious^a^ | not serious | not serious | serious^b^ | none | 168 | 128 | - | MD **17.83 days fewer** (24.91 fewer to 10.75 fewer) | ⨁◯◯◯ Very low | CRITICAL |
| **Percentage of participants returned to work or employed (follow-up: range 6 weeks to 12 months)** | | | | | | | | | | | | |
| 8 | randomised trials^c^ | very serious^d^ | serious^e^ | not serious | serious^f^ | none | 426/561 (75.9%) | 375/499 (75.2%) | **RR 1.03** (0.91 to 1.18) | **23 more per 1,000** (from 68 fewer to 135 more) | ⨁◯◯◯ Very low | CRITICAL |
| **Days of sick leave (follow-up: range 4 months to 12 months)** | | | | | | | | | | | | |
| 6^g^ | randomised trials | serious^h^ | serious^i^ | not serious | not serious | publication bias strongly suspected^j^ | 2254 | 1707 | - | MD **3.27 days lower** (8.1 lower to 1.56 higher) | ⨁◯◯◯ Very low | CRITICAL |
| **Percentage of participants with sick leave (follow-up: range 2 weeks to 12 months)** | | | | | | | | | | | | |
| 9^k^ | randomised trials^l^ | very serious^m^ | not serious | not serious | serious^n^ | publication bias strongly suspected^j^ | 173/585 (29.6%) | 140/458 (30.6%) | **RR 1.06** (0.82 to 1.36) | **18 more per 1,000** (from 55 fewer to 110 more) | ⨁◯◯◯ Very low | CRITICAL |
| **Returning to full or normal duties (follow-up: range 6 weeks to 7 months)** | | | | | | | | | | | | |
| 4 | randomised trials^o^ | very serious^p^ | not serious | not serious | serious^q^ | none | 138/171 (80.7%) | 96/144 (66.7%) | **RR 1.17** (1.01 to 1.36) | **113 more per 1,000** (from 7 more to 240 more) | ⨁◯◯◯ Very low | IMPORTANT |

**CI:** confidence interval; **MD:** mean difference; **RR:** risk ratio

**Explanations**

a. 2 studies had high risk of bias (1 or 2 serious risk categories) and 1 study was rated as some concerns

b. Optimal Information Size not met as sample size too small

c. 3 studies were non-RCTs

d. 7 out of 8 studies had a high risk of bias (1 or 2 serious risk categories)

e. There was significant heterogeneity (68%). Some confidence intervals did not overlap.

f. Optimal Information Size not met as sample size too small to detect small effect (~RR = 1.20), assuming control group risk is 75%, with 80% power.

g. 6 studies, 7 comparisons

h. 3 studies at high risk of bias, 3 studies had some concerns

i. There was significant heterogeneity (I2 = 60%)

j. Possible publication bias detected when examining the funnel plot.

k. 9 studies, 10 comparisons

l. 2 studies were non-RCTs

m. 7 studies at high risk of bias (1 or 2 serious risk categories), 2 studies had some concerns

n. Optimal Information Size not met as sample size too small to detect small effect (~RR=1.20), assuming control group risk is 30%, with 80% power.

o. 2 studies were non-RCTs

p. All studies had a high risk of bias using the ROB2 and ROBINS tools

q. The lower confidence interval (-0.19) represents a small effect that interventions worsen the likelihood of returning to full or normal duties after RTC-related musculoskeletal injury. The upper confidence interval (0.31) represents a small to medium effect that interventions improve the likelihood of returning to full or normal duties after RTC-related musculoskeletal injury.

**Paper:** Effectiveness of interventions on work outcomes after road traffic crash-related musculoskeletal injuries: a systematic review and meta-analysis, submitted to Journal of Occupational Rehabilitation

**Authors**: Charlotte L. Brakenridge, Esther J. Smits, Elise M. Gane, Nicole E. Andrews, Gina Williams, Venerina Johnston

**Contact:** Charlotte L. Brakenridge, [c.brakenridge@uq.edu.au](mailto:c.brakenridge@uq.edu.au), The University of Queensland, School of Human Movements and Nutrition Sciences, Brisbane, QLD, Australia
